# Single-Cell Analysis of Different Stages of Oral Cancer Carcinogenesis in a Mouse Model

**DOI:** 10.3390/ijms21218171

**Published:** 2020-10-31

**Authors:** Ling-Yu Huang, Yi-Ping Hsieh, Yen-Yun Wang, Daw-Yang Hwang, Shih Sheng Jiang, Wen-Tsung Huang, Wei-Fan Chiang, Ko-Jiunn Liu, Tze-Ta Huang

**Affiliations:** 1 Institute of Clinical Medicine, College of Medicine, National Cheng Kung University, Tainan 701401, Taiwan; helen85116@gmail.com; 2 Institute of Basic Medical Sciences, College of Medicine, National Cheng Kung University, Tainan 701401, Taiwan; pin1000822@gmail.com; 3 School of Dentistry, College of Dental Medicine, Kaohsiung Medical University, Kaohsiung 80708, Taiwan; winnie30304@hotmail.com; 4 National Institute of Cancer Research, National Health Research Institutes, Tainan 70456, Taiwan; dawyanghwang@nhri.edu.tw (D.-Y.H.); ssjiang@nhri.edu.tw (S.S.J.); kojiunn@nhri.edu.tw (K.-J.L.); 5 Chi Mei Medical Center, Liouying, Tainan 73659, Taiwan; huangwentsung@mac.com (W.-T.H.); bigfanfan@yahoo.com.tw (W.-F.C.); 6 School of Dentistry, National Yang Ming University, Taipei 11221, Taiwan; 7 Graduate Institute of Medicine, College of Medicine, Kaohsiung Medical University, Kaohsiung 807377, Taiwan; 8 Institute of Clinical Pharmacy and Pharmaceutical Sciences and Institute of Clinical Medicine, National Cheng Kung University, Tainan 704302, Taiwan; 9 School of Medical Laboratory Science and Biotechnology, Taipei Medical University, Taipei 110301, Taiwan; 10 Institute of Oral Medicine, Department of Dentistry, Division of Oral and Maxillofacial Surgery, Department of Stomatology, National Cheng Kung University Hospital, College of Medicine, National Cheng Kung University, Tainan 701401, Taiwan

**Keywords:** mouse model, carcinogenesis, single-cell RNA sequencing, oral cancer

## Abstract

Oral carcinogenesis involves the progression of the normal mucosa into potentially malignant disorders and finally into cancer. Tumors are heterogeneous, with different clusters of cells expressing different genes and exhibiting different behaviors. 4-nitroquinoline 1-oxide (4-NQO) and arecoline were used to induce oral cancer in mice, and the main factors for gene expression influencing carcinogenesis were identified through single-cell RNA sequencing analysis. Male C57BL/6J mice were divided into two groups: a control group (receiving normal drinking water) and treatment group (receiving drinking water containing 4-NQO (200 mg/L) and arecoline (500 mg/L)) to induce the malignant development of oral cancer. Mice were sacrificed at 8, 16, 20, and 29 weeks. Except for mice sacrificed at 8 weeks, all mice were treated for 16 weeks and then either sacrificed or given normal drinking water for the remaining weeks. Tongue lesions were excised, and all cells obtained from mice in the 29- and 16-week treatment groups were clustered into 17 groups by using the Louvain algorithm. Cells in subtypes 7 (stem cells) and 9 (keratinocytes) were analyzed through gene set enrichment analysis. Results indicated that their genes were associated with the MYC_targets_v1 pathway, and this finding was confirmed by the presence of cisplatin-resistant nasopharyngeal carcinoma cell lines. These cell subtype biomarkers can be applied for the detection of patients with precancerous lesions, the identification of high-risk populations, and as a treatment target.

## 1. Introduction

Oral cancer is currently one of the most common cancers worldwide, and its incidence and mortality are increasing annually [1]. Its major risk factors are smoking cigarettes, drinking alcohol and chewing betel nut; other factors include excessive sun exposure, viruses, fungal infections, poor diet, poor oral hygiene, inappropriate dentures (leading to chronic inflammation of the oral cavity), immunodeficiency, and genetic polymorphism among individuals of different races [2]. Oral cancer is mainly treated through surgical resection, whereas radiotherapy, chemotherapy, and immunotherapy are used as postoperative adjunctive therapy for patients with more advanced cancer [3]. However, these treatments have limited therapeutic effects on patients with advanced cancer. Therefore, identifying key factors for tumor cell subtypes that predict the progression of precancerous lesions into cancer is essential for developing treatment for preventing the rapid carcinogenesis of tumor cells.

Head and neck cancer studies have used numerous cancer cells to explore gene expression and regulation [4,5]. However, human tumor cells are composed of malignant, immune, and stromal cells. The gene expression of an important subtype of cancer cells can easily be overlooked when differences in the expression of all cells are being averaged. Single-cell analysis can be used to distinguish between genetic and nongenetic mechanisms, identify characteristics of the tumor microenvironment and cell interactions, determine the likelihood of disease recurrence, identify minimal residual disease and rare tumor cell subpopulations, explore cell sources and influence pathways, and identify cell subpopulations that significantly affect disease progression [6].

Current approaches for separating single cells can be categorized as micromanipulation, flow cytometry for fluorescence-activated cells, laser capture microdissection, and microfluidics. Micromanipulation requires the use of a microscope to absorb cells for follow-up research but requires the application of advanced operating techniques and freshly harvested cells. Flow cytometry sorting can be used for cell calibration and enables the simultaneous screening of a large number of target cells; however, because it requires numerous input samples and its high-speed vibration and high voltage cause cells to form charged droplets, which may affect cell viability, flow cytometry is unsuitable for samples that are rare or difficult to obtain. Laser microscopy can be used for fixed tissues or frozen sections; through a special microscope, a laser is used to cut cells, thus revealing the position of cells in the tissue. However, this approach requires advanced operation technology and low productivity and is prone to cell contamination. Microfluidic technology allows a group of cells to sequentially pass through an aperture, where each cell, the required reagent (reverse transcribed into cDNA), and an identifier are contained in a drop. Commercial platforms for single-cell isolation include the high-throughput Chromiun System (10x Genomics. Pleasanton, CA, USA), Nadia Instrument (Dolomite Bio. Royston, Hertfordshire, UK), InDrop System (1CellBio. Watertown, MA, USA), Illumina Bio-Rad ddSEQ Single-Cell Isolator, Tapestri Platform (MissionBio. San Francisco, CA, USA), BD Rhapsody Single-Cell Analysis System (BD. Franklin Lakes, NJ, USA), medium-throughput ICELL8 Single-Cell System (Takara. Mountain View, CA, USA), and C1 System (Fluidigm. South San Francisco, CA, USA) [7].

Single-cell RNA sequencing (scRNA-seq) is a powerful approach. Current applications for scRNA-seq include identification of the cell composition of tissues, understanding how cells develop from stem and progenitor cells, analysis of the clonal evolution of cancer, understanding cell communication through cell receptor–ligand networks, and understanding the cellular response to genetic manipulation or drugs [8]. In cancer research, scRNA-seq was applied for defining T-cell receptor sequences in individual cells, which is pivotal for identifying tumor-specific neoantigens present in the major histocompatibility complex in tumor cells [9]. The application of scRNA-seq on cancerous and immune cells from patients with melanoma has revealed T-cell exhaustion signatures and their association with T-cell activation [9], which are the distinctive functional composition of T cells in hepatocellular carcinoma [10]. It has also identified two major subsets of cells characterized by epithelial and stromal gene expression patterns attributable to the proliferation of genes, including genes associated with oxidative phosphorylation and MYC activity in ovarian cancer [11], as well as differences between isocitrate dehydrogenase mutants astrocytoma and oligodendroglioma in distinct tumor microenvironments and their signature genetic events. Both tumor types share similar developmental hierarchies and lineages of glial differentiation [12]. Moreover, for head and neck squamous cell carcinoma, subtypes are characterized by their malignant and stromal composition, and p-EMT has been established as an independent predictor of nodal metastasis, grade, and adverse pathologic features [13].

Oral carcinogenesis is the sequential development of the normal oral mucosa into potentially malignant disorders and finally into oral cancer. In clinical practice, the carcinogenesis of cell subtypes cannot be analyzed in the same patient because of ethical concerns that we cannot let the patient’s precancerous lesion develop into cancer without any intervention. The variations that exist among individual patients also make it difficult to compare the carcinogenesis sequence of cell subtypes between individuals. These concerns limit the analytic power of scRNA-seq. A mouse model with arecoline and 4-nitroquinoline 1-oxide (4-NQO) cotreatment, which mimics the etiology of oral cancer in humans, was utilized to better investigate oral cancer carcinogenesis [14]. Mice were exposed to drinking water with 4-NQO (200 μg/mL) and arecoline (500 μg/mL) and were sacrificed at 8, 16, 20, or 29 weeks. Except for the mice treated for 8 weeks and sacrificed at 8 weeks, all were treated for 16 weeks and then given water for the remaining weeks until sacrificed. Lesions on the tongue were excised for analysis. The animal model applied in this research minimized individual heterogeneity and thus, it can be used during the analysis of different carcinogenesis cell subtypes in different patients.

Suitable biomarkers must be identified for detecting oral mucosa cells that have gradually formed precancerous lesions and preventing them from evolving into malignant cancer cells. In clinical treatment, patients are targeted on the basis of the average expression of all genes in a whole tissue. However, an increasing number of studies have confirmed that the entire tumor ecosystem is highly complex and consists of a variety of cell groups with varying abilities to worsen disease progression. Through scRNA-seq gene detection and analysis, a detailed and holistic understanding of the entire cell population and different cell subpopulations can be obtained, and the primary factor influencing the survival of patients with tumor progression and metastasis can be effectively identified.

## 2. Results

### 2.1. Carcinogenesis in the Experimental Animal Model

**Figure 1:**
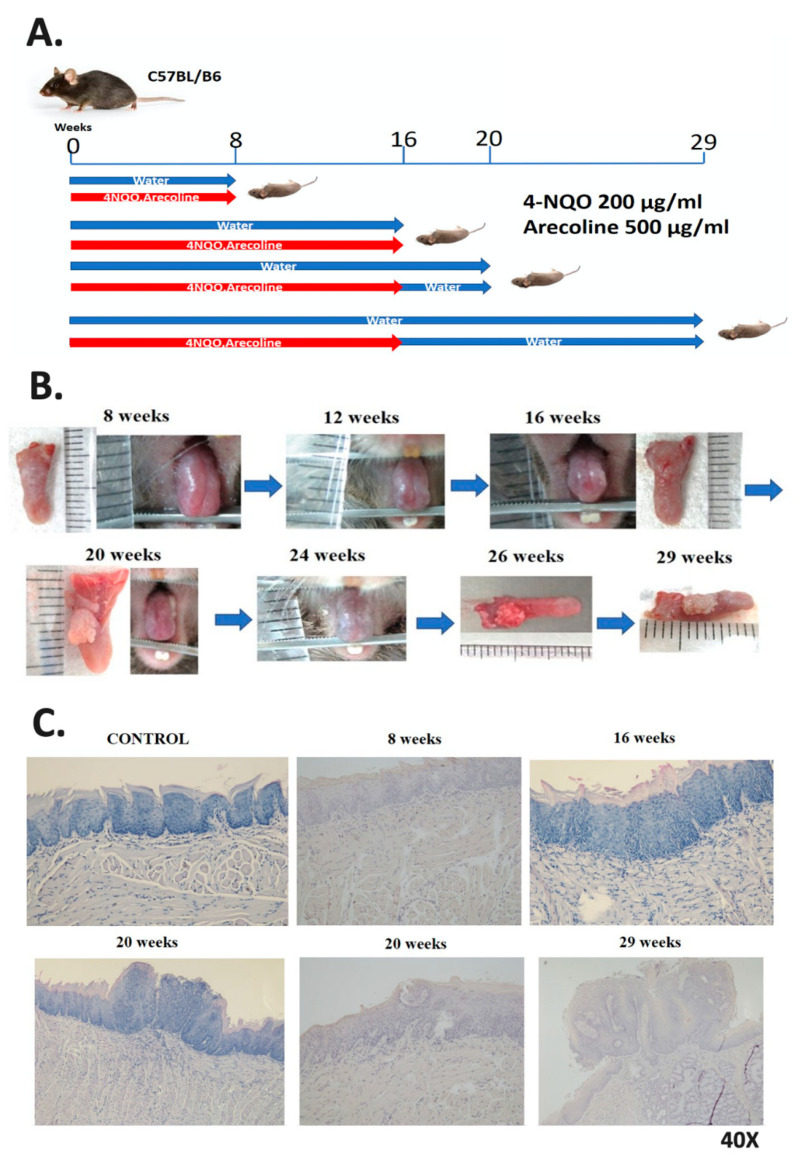
The carcinogen 4-nitroquinoline 1-oxide (4-NQO) (similar to carcinogens found in cigarettes) and arecoline (the carcinogen found in betel nut) were used to induce head and neck cell carcinogenesis in mice. (**A**) Four experimental and control groups were examined. Mice in the experimental group were fed drinking water containing 4-NQO and arecoline and then monitored until being sacrificed. (**B**) The progression of precancerous lesions on the tongue to cancer was monitored when mice were alive, and they were sacrificed at different time points. With the increasing duration of treatment in the experimental group, white lesions began to appear on the tongues of mice. Tumors were observed on the tongues of mice at week 16 and had become invasive carcinoma by week 29. (**C**) The tongues of mice in the control and experimental groups underwent hematoxylin and eosin staining for histopathological examination.

In this study, 4-NQO (200 μg/mL) and arecoline (500 μg/mL) were given to mice in their drinking water every day to simulate the carcinogenic effects of cigarettes and betel nut in transforming healthy oral mucosa cells into precancerous lesions and eventually malignant lesions (Figure 1A). Figure 1B displays changes in the tongues of mice. In terms of their gross appearance, the tongues exhibited white lesions after 16 weeks of treatment. Hematoxylin and eosin (H&E) staining results revealed squamous hyperplasia of tongue cells after 16 weeks of treatment (Figure 1C), tongue tumors (Figure 1B) and tongue squamous cell carcinoma (Figure 1C) after 16 weeks of treatment, and 4 weeks of follow-up and invasive tumor formation at the end of the experimental period (Figure 1B). H&E staining results at 29 weeks revealed advanced tongue squamous cell carcinoma (Figure 1C). Tongue lesions at 16 and 29 weeks were excised for scRNA-seq, RNA, and protein expression analyses. The results indicated the poor differentiation of squamous cell carcinoma at 16 weeks and advanced squamous cell carcinoma at 29 weeks.

### 2.2. scRNA-seq in Control and Experimental Groups

In this study, mouse tongues were excised for scRNA-seq analysis at 16 and 29 weeks. Each cell was wrapped in oil droplets to generate GEMs (Supplementary Materials Figure S1). Inside the GEMs, intracellular mRNA was reverse transcribed into cDNA, and Qubit fluorometric quantitation (Thermo Fisher Scientific) was used for the concentration measurement. The data were as follows: 0.162, 0.501, and 0.716 ng/μL for the 16-week control group; 0.126, 0.446, and 0.584 ng/μL for the 16-week experimental group; 0.132 and 3.100 ng/μL for the 29-week control group, and 0.288, 5.340, and 5.280 ng/μL for the 29-week experimental group. The required number of cycles in the library construction was according to its concentration. After library construction, the concentrations were 33.8, 19.0, and 25.0 ng/μL for the 16-week control group; 32.0, 14.0, and 18.0 ng/μL for the 16-week experimental group; 21.0 and 14.0 ng/μL for the 29-week control group, and 37.6, 23.4, and 26.8 ng/μL for the 29-week experimental group. The 11 groups of samples were sequenced for next-generation sequencing analysis, and the results were mapped with the mouse genome mm10 and divided into 16- and 29-week control and experimental groups. All cell reads are presented in Table 1. The total number of sequencing reads varied according to the number of cells packed each time, and the mouse genome (mm10) accounted for more than 92% of the map.

**Figure 2:**
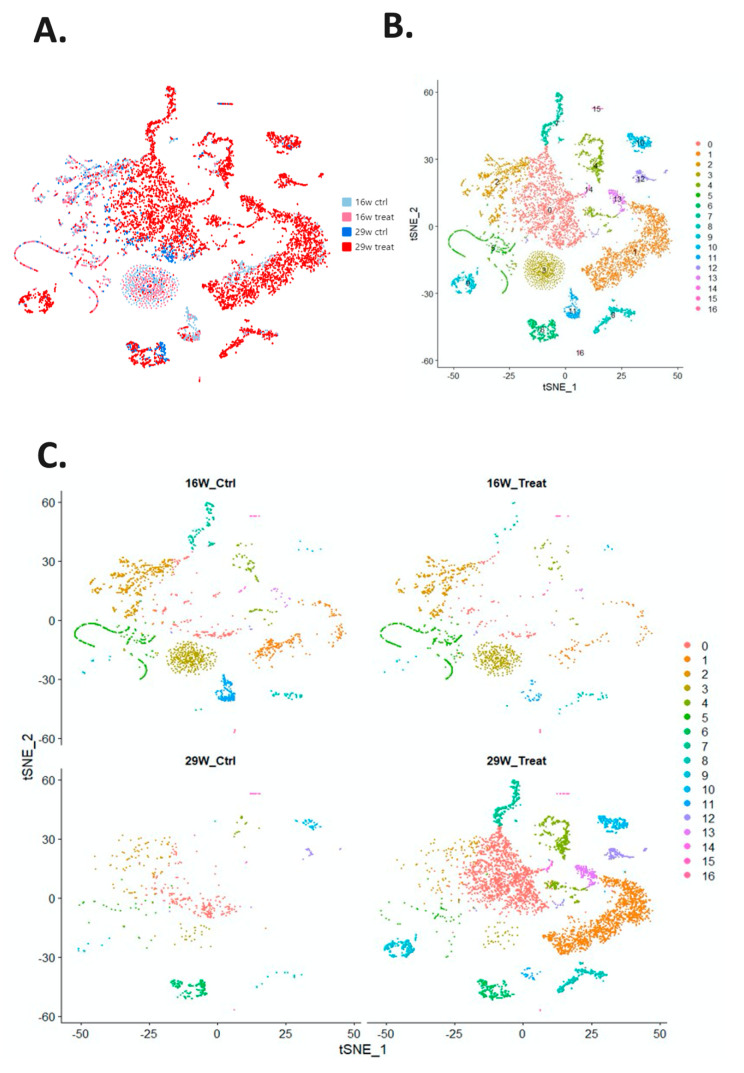

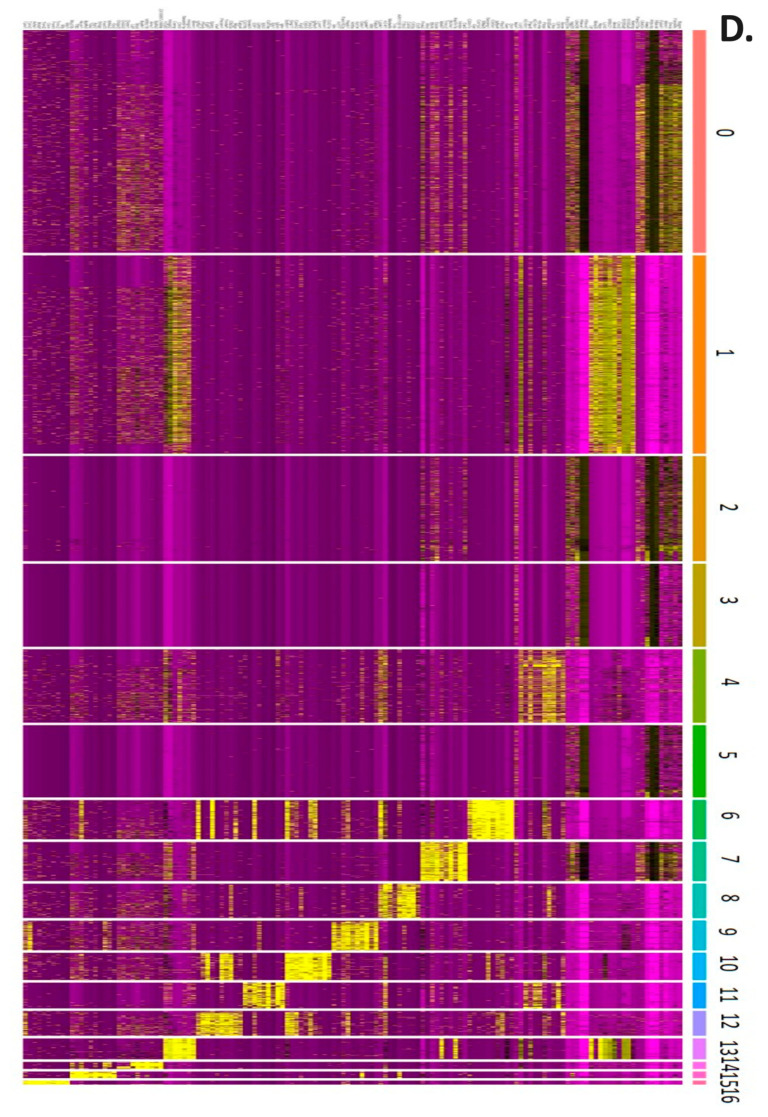
Visual distribution of dimensionality reduction in cell populations. (**A**) The cell distribution of all samples generated through t-distributed stochastic neighbor embedding and t-random neighbor embedding, with four groups as the source (16- and 29-week control and experimental groups). (**B**) Cells divided into 17 subtypes by using the Louvain algorithm. (**C**) Separation of the four major groups of cell populations and the distribution of the 17 cell subtypes. (**D**) Heat map of the 17 cell subtypes. After cells were separated into 17 subtypes, the 10 genes with the highest expression in each subtype of cells were identified and compared between subtypes. Yellow indicates that the gene expression is higher than in other cell subtypes.

The Cellranger (v3.1.0, 10x Genomics) was used to analyze the sequencing reads for each sample, and the results of mapping with the mouse reference genome (mm10). The results of all samples were aggregated, and corrections were made on the basis of the library size of each sample. Each cell had read counts of 31,053 genes, which were analyzed using the Seurat R package and normalized according to the total count for each cell. Genes with the greatest differences between cells were identified; 1000 genes were selected from 31,053 genes for subsequent analysis (Supplementary Materials Figure S2a), and then, principal component analysis was performed to linearly reduce the dimensionality of 1000 gene data (Supplementary Materials Figure S2b,c). The first 10 principal components were used to calculate clustering and dimensionality reduction and provide data visualization of t-distributed stochastic neighbor embedding (tSNE) (Figure 2A,B). The cells were clustered into 17 subtypes. The cells in the four groups (16- and 29-week control and experimental groups) were plotted separately with tSNE (Figure 2C). The top 10 genes with the highest expression compared with other cell populations were identified and represented by a clustering heat map (Figure 2D). Most cell subtypes had a high expression of specific genes. The genes corresponding to each cell subtype in the cluster heat map are listed in Supplementary Materials Table S5.

**Figure 3:**
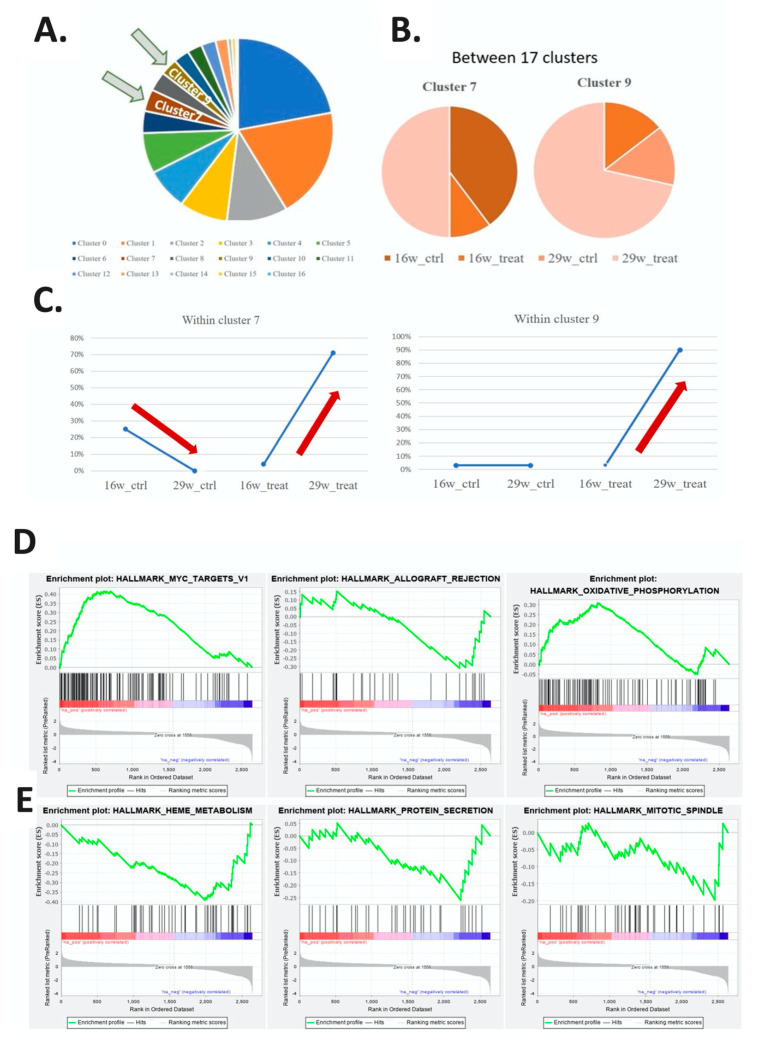

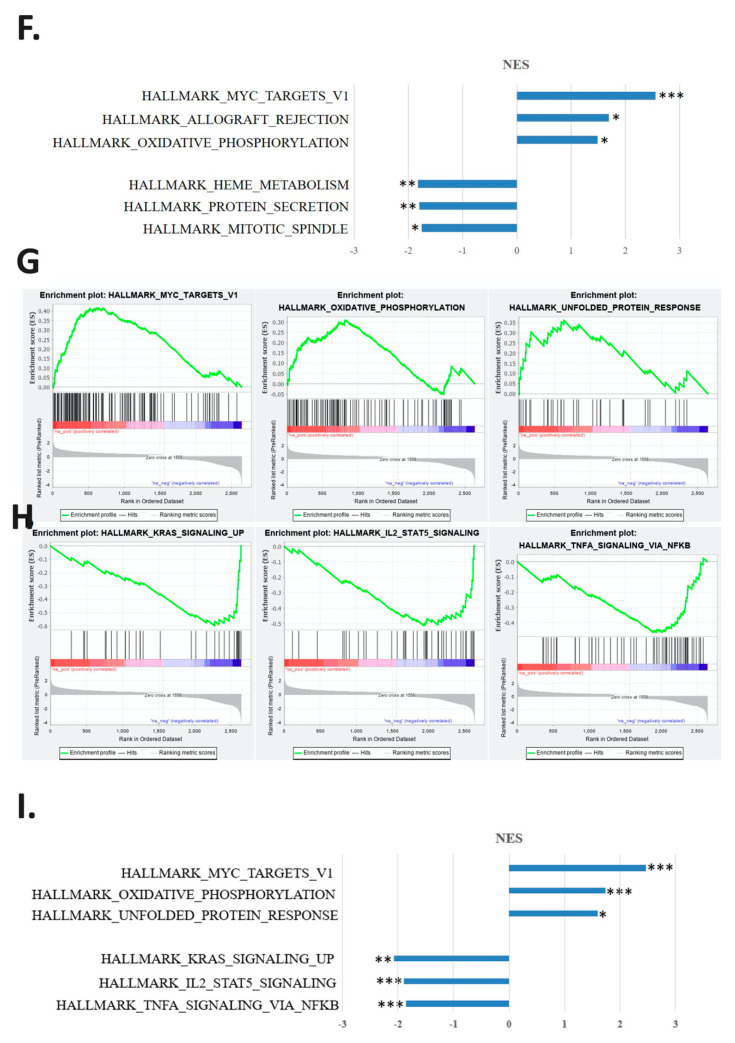

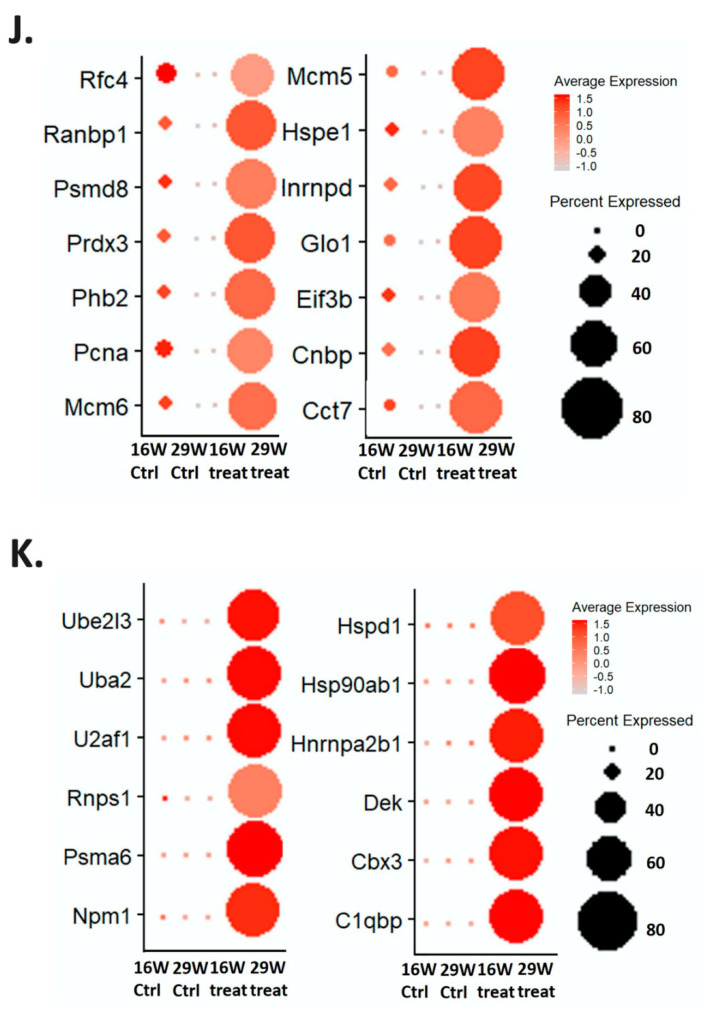
Comparison of the compositions of cell subtypes between the 16- and 29-week control and experimental groups. (**A**) Proportion of the 17 cell subtypes with respect to the total cell composition presented as a pie chart. (**B**) Comparison of the proportion of the seventh and ninth cell subtypes in the four major groups. (**C**) Line graph comparing the proportions of the seventh and ninth cell subtypes in the four major groups. The proportion increased with increasing treatment duration in in the experimental group. (**D**–**F**) Gene function enrichment analysis of the seventh cell subtype. (**D**) Line chart indicating the three most significant pathways involved in the increase in gene expression in the 16- and 29-week experimental groups. (**E**) Line chart showing the three most significant pathways involved in the decline in gene expression in the 16- and 29-week experimental groups. (**F**) Bar graph of the normalized enrichment scores (NESs) and *p* values of these three paths (* *p* < 0.05; ** *p* < 0.01; *** *p* < 0.001). (**G**–**I**) Gene function enrichment analysis of the ninth cell subtype. (**G**) Line chart indicating the three most significant pathways involved in the increase in gene expression in the 16- and 29-week experimental groups. (**H**) Line chart showing three most significant pathways involved in the decline in gene expression in the 16- and 29-week experimental groups. (**I**) Bar graph displaying the calculated NESs and *p* values of these three paths (* *p* < 0.05; ** *p* < 0.01; *** *p* < 0.001). (**J**) Dot diagram of genes involved in the MYC_targets_v1 pathway in the seventh subtype. The average gene expression level and the percentage of cells in the four groups are indicated by the color and size of dots. The average expression level and cell ratio of genes in the 29-week experimental group were significantly higher than those in the other groups. (**K**) Dot diagram of genes involved in the MYC_targets_v1 pathway in the ninth subtype. The average gene expression level and the percentage of cells in the four groups are indicated by the color and size of dots. The average expression level and cell ratio of genes in the 29-week experimental group were significantly higher than those in the other groups.

The tSNE map offered a comparison of the control and experimental groups with respect to the proportion of a subtype of cells within and between cell groups (Table 2). The proportions for each cell subtype are displayed in a pie chart (Figure 3A). The proportions of the seventh and ninth cell subtypes in each group are displayed in Figure 3B. The proportion of the seventh cell subtype was lower in the 29-week control group than in the 16-week control group, and that of the ninth cell subtype was similar for both of these groups. The proportions of the seventh and ninth cell subtypes were higher in the 29-week experimental group than in the 16-week experimental group (Figure 3C). The seventh cell subtype was catalogued as stem cell and the ninth subtype was catalogued as keratinocyte by PanglaoDB [15]. The results revealed increased oncogenic activity in these two cell subtypes. Gene set enrichment analysis was performed to determine the regulatory pathways involved in carcinogenesis.

### 2.3. Regulatory Pathways Involved in the Carcinogenesis of a Mouse Animal Model

The tSNE map revealed that the seventh and ninth cell subtypes exhibited an increasing trend in the proportion of cells in the experimental group; their differences in expression genes at 29 and 16 weeks in the experimental group are listed in Supplementary Materials Table S6. Analysis of the molecular signature database (MSigDB) hallmark gene set collection was performed to identify three relevant regulatory pathways for the expression level of different genes in the seventh cell subtype in the 16- and 29-week experimental groups. The increasing expression pathways were MYC_targets_v1, Allograft_rejection, and Oxidative_phosphorylation, as represented by an enrichment plot (Figure 3D); the decreasing expression pathways were Heme_metabolism, Protein_secretion, and Mitotic_spindle (Figure 3E). The calculation method is based on the fold change of all genes in the 16- and 29-week experimental groups. A line graph of enrichment scores obtained by the cells of different genes was plotted for a comparison of the selected gene group data within the range between the maximum and minimum peak position; the gene groups at the left and right end points of the graph (leading-edge subset) are important genes involved in regulating the pathway. The normalized enrichment scores (NESs) of the regulatory pathways ranged between −2 and 3, and the calculated *p* value was significant (Figure 3F) (Supplementary Materials Table S7).

In the ninth cell subtype, an enrichment plot was generated to display the top three related regulatory pathways with the greatest increase and the top three with the greatest decrease in gene expression levels between the 16- and 29-week experimental groups. Those with the greatest increase were MYC_targets_v1, Oxidative_ phosphorylation, and Unfolded_protein_response (Figure 3G); those with the greatest decrease were KRAS_signaling_up, IL2_STAT5_signaling, and TNFα_signaling_via_NFkB (Figure 3H). The NESs of the first three regulatory pathways ranged between −3 and 3, and the *p* value was significant (Figure 3I) (Supplementary Materials Table S8).

The gene expression clusters of the most significant regulatory pathways of the seventh and ninth cell subtypes in the 16- and 29-week experimental groups were MYC_targets_v1, as represented by incremental points in Figure 3J,K. For the seventh and ninth cell subtypes, the average expression of the most highly expressed genes in the MYC_targets_v1 pathway were increased, and the proportion of cells that expressed these genes was also increased in the 29-week experimental group compared with the 16-week experimental group. The ratio of the average expression among cell expression of these genes exhibited a downward trend in the 29-week experimental group compared with the 16-week experimental group.

### 2.4. Validation of the Gene Expression in the Regulatory Pathways in Cisplatin-Resistant Cell Lines

**Figure 4:**
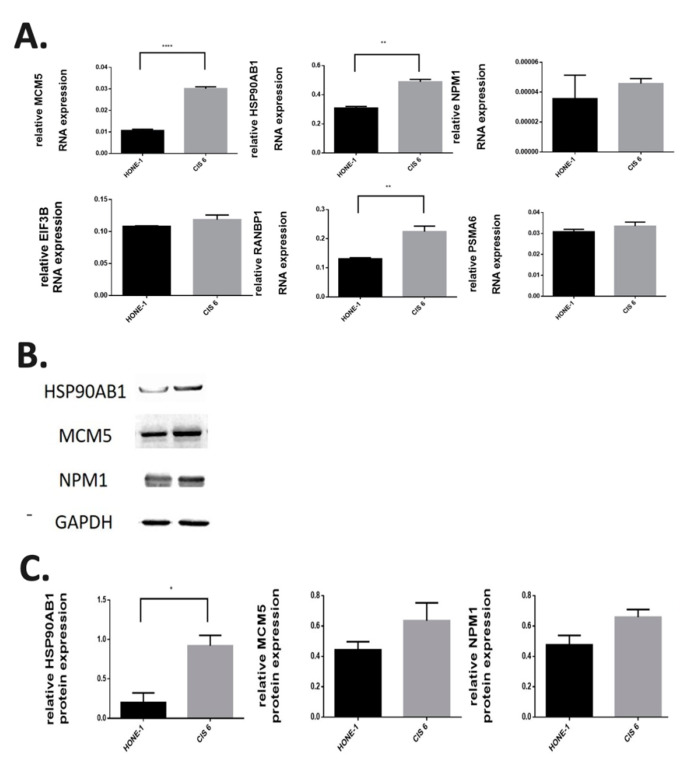
RNA and protein expression in cisplatin-resistant nasopharyngeal carcinoma cell lines. (**A**) Expression of MCM5, HSP90AB1, NPM1, EIF3B, RANBP1, and PSMA6 RNA analyzed through real-time quantitative polymerase chain reaction in the nasopharyngeal carcinoma cell line HONE-1 and the cisplatin-resistant nasopharyngeal carcinoma cell line HONE1-CIS6. Expression levels were higher in the HONE1-CIS6 cell line than in the nasopharyngeal carcinoma cell lines (** *p* < 0.005; **** *p* < 0.0001). (**B**) Protein expression levels of HSP90AB1, MCM5 and NPM1 analyzed in HONE-1 and HONE1-CIS6 through Western blotting; HONE1-CIS6 had the highest protein expression levels (* *p* < 0.05). (**C**) Relative quantitative protein expression levels of HSP90AB1, MCM5 and NPM1 to GAPDH in HONE-1 and HONE1-CIS6.

The involvement of the genes RANBP1, MCM5, EIF3B, PSMA6, NPM1, and HSP90AB1 in the most significant regulatory pathways of the seventh and ninth cell subtypes was further confirmed in the nasopharyngeal carcinoma cell line (HONE-1), and cisplatin was used to screen for the expression of these genes in the drug-resistant HONE1-CIS6 cell line. RT-PCR results revealed that these genes were more highly expressed in the drug-resistant HONE1-CIS6 cell line than in the HONE-1 cell line (Figure 4A). Additionally, these genes had higher protein expression levels in the drug-resistant HONE1-CIS6 cell line than in the HONE-1 cell lines (Figure 4B) (Supplementary Materials Figure S4).

## 3. Discussion

Oral cancer is the sixth most common cancer among men worldwide [16]. In Taiwan, it ranks fifth in mortality among all cancers in the general population and fourth in the male population [17]. Surgery is currently the primary approach for treating oral cancer. For advanced stages, chemotherapy, radiation, and immune therapies are the primary treatment options. However, the efficacy of these treatments is limited when the diagnosis is made at a later stage [18]. Therefore, identifying new biomarkers for detecting precancerous lesions and preventing their progression to oral cancer are pivotal.

The composition of cancer cells is diverse and complex in all human cancers. In the same tumor, cells may vary in terms of their genetic, epigenetic, and transcription characteristics and proteomic dysregulation, all of which affect the overall disease progression. If evaluations are based on the characteristics of total cells, then the result may be failure to apply a treatment modality that can effectively target the most oncogenic tumor cell subtype, thus considerably reducing the therapeutic effects and patients’ likelihood of survival [12,19]. For example, some cancer cell subtypes have a high degree of proliferation, and some subtypes are more invasive and metastatic. The entire tumor microenvironment is highly heterogeneous, and higher heterogeneity among cells corresponds to weaker therapeutic effects, resistance to disease treatments, and an increased risk of recurrence [12]. Traditional RNA expression analysis is often employed to study the characteristics of a group of cells by averaging the characteristics displayed by specific cell subtypes. The use of single-cell technology provides a new approach for exploring the entire tumor microenvironment and clarifying the functions of individual cells. For triple negative breast cancer, which is the most aggressive and chemotherapy-resistant type of breast cancer, scRNA-seq can be used to identify specific cells that induce cancer formation and affect disease progression [20]. Single-cell technology can also be used to elucidate the mechanisms of cell resistance and genomic and transcription dysregulation [21]. In ovarian cancer, malignant cells and cancer-related fibroblasts express genes related to the JAK–STAT signaling pathway, and inhibiting this pathway was reported to promote effective anti-tumor ability in the primary cell cultures and xenograft models of patients [22]. Tumor-infiltrating lymphocytes are a potential benefit of immunotherapy and enhance clinical response to cancer, and analysis of the T-cell population in hepatoma tissues revealed that the *LAYN* gene in infiltrating T cells can inhibit the function of regulatory T cells and CD8 T cells [10]. The use of scRNA-seq technology can enable the further exploration of factors affecting the course of a disease and reveal the characteristics and control pathways of specific cell subtypes, thus facilitating disease prevention and detection and improving treatment strategies to prevent disease progression or recurrence. In this research, scRNA-seq was used to investigate the different stages of carcinogenesis in an oral cancer mouse model and revealed associations of oncogenic clusters 7 (stem cells) and 9 (keratinocytes) with the MYC_targets_v1 pathway.

Similar to previous research [23,24], an experiment was conducted in the present study wherein 4-NQO, which is a soluble carcinogen in cigarettes, was added to the drinking water of mice in the experimental group. Carcinogens in water can disrupt DNA and induce the production of reactive oxygen species. The other carcinogen used in this study, arecoline, is the main alkaloid in betel nut. It is known to have cytotoxicity and genotoxicity and induce mutations, which can cause histological or other biological changes [25]. In mice, the combination of the two drugs can mimic the occurrence of oral cancer, ranging from epithelial cell hyperplasia and cell dysplasia to invasive squamous cell carcinoma [14]. H&E staining and images captured every week confirmed the carcinogenesis of the mice tongues. The findings regarding the carcinogenesis observed in the male C57BL/6J mouse model in this study can also serve as a reference for minimizing the effects of variance among individual patients during analysis of carcinogenesis in different cell subtypes.

In this study, 10x Chromium single-cell technology was used to extract healthy-tongue and tumor cells in the 16- and 29-week control and experimental groups for single-cell sorting. The factors of carcinogenesis that caused the cells to become specific malignant cell subtypes were analyzed in four groups (16- and 29-week control and experimental groups). The cells were divided into 17 subtypes, and two cell subtypes with little difference in the proportion or expression level between the 16- and 29-week control groups were selected; the cell proportion and expression exhibited significant upregulation in the 16- and 29-week experimental groups. These two cell subtypes were inferred to be influential oncogenic factors in carcinogenesis. Gene function enrichment analysis [26,27] revealed that the most significant regulatory pathway involved in the two cell subtypes is MYC_targets_v1. *MYC* is not only a regulatory gene that affects gene expression but also a proto-oncogene. Its three related genes, *c-myc* (*MYC*), *l-myc* (*MYCL*), and *n-myc* (*MYCN*), have been found to be involved in many human cancers, with the regulation of some signal pathways leading to carcinogenesis [28]. Additionally, *c-myc* is involved in some physiological functions, including cell cycle regulation, ribosome biogenesis, protein synthesis, stem cell function regulation, and cell apoptosis [29]. *MYC* mutations have been related to Burkitt’s lymphoma and leukemia [30,31]; they are activated through gene rearrangement and gene amplification, which affect other genes. In addition, the overexpression of the *MYC* gene is also found in breast, cervical, colorectal, lung, and gastric cancers and leads to cell proliferation [32,33,34,35,36]. The overexpression of the *MYC* gene has also been noted in most patients with oral cancer. For patients with T3 or T4 oral cancer, *MYC* gene expression is related to the likelihood of survival and can serve as a key biomarker for treatment [37].

In the seventh cell subtype (stem cells) involved in the MYC_targets_v1 pathway, increased *RANBP1*, *MCM5*, and *EIF3B* gene expression was noted in the 29-week experimental group; the average expression level and cell proportion noticeably increased. The regulation of *c-Myc* mRNA is accompanied by specific RNA-binding proteins and also occurs at the translation level. For example, *CELF1* inhibits Myc translation by reducing the binding of *c-Myc* mRNA to *HuR*, and it provides new insights into the molecular function of RNA-binding proteins [38]. The interaction between RNA-binding proteins and c-Myc mRNA may become a new target for cancer treatment [39]. *MCM5* is a DNA replication licensing factor. The protein is upregulated during the transition from G0 to G1/S of the cell cycle, and it is involved in numerous *MYC* regulatory pathways related to DNA replication [40]. *EIF3B* is a member of the eukaryotic translation initiation factor 3 subunit B and is related to the transcription initiation of cancer-related genes. Gene function enrichment analysis revealed that *EIF3B* is involved in the regulatory pathway and overexpressed in patients with hepatoma [41].

The ninth cell subtype (keratinocytes) had a larger proportion of cells and increased *PSMA6*, *NPM1*, and *HSP90A* expression in the 29-week experimental group. *PSMA6* is a gene encoding the proteasome α6 subunit protein, which can assemble the 20S proteasome complex, and the proteasome is a key component of the ubiquitin–proteasome system [42]. This system has an important function in malignant transformation and regulates gene expression by degrading transcription factors, such as *c-Myc*. The ubiquitin–proteasome system participates in the development of many malignant cancers [43]. Nucleophosmin is a highly expressed protein located in the nucleolus that moves between the nucleus and cytoplasm. Its main functions are ribosome maturation and export, participation in centrosome replication, cell cycle progression, histone assembly, and response to various stimuli. In acute myeloid leukemia and some solid tumors, *NPM1* gene overexpression is related to mitotic index and metastasis [44]. The Cancer Genome Atlas database was used to analyze the relationship between *NPM1* mRNA expression and the survival rate of patients with head and neck cancer. High expression of *NPM1* is related to a lower likelihood of survival (Supplementary Materials Figure S3). Nucleophosmin is crucial to the oncogenic activity of *c-Myc*. Overexpression of nucleophosmin stimulates *c-Myc* to induce hyperproliferation and transformation [45]. Subtypes of heat shock protein 90 include HSP90AA (encoded by *HSP90AA1* and *HSP90AA2*) and HSP90AB (encoded by *HSP90AB1*). Studies have demonstrated that *c-Myc* directly activates the transcription of *HSP90A*, and this transcriptional induction occurs in different tissues and is not related to cell proliferation. Since the *c-Myc* binding site is near the *HSP90A* gene promoter, *c-Myc* induces *HSP90A* expression and can control many message transmission pathways, including cell transformation [46].

The drug-resistant human nasopharyngeal carcinoma cell line HONE1-CIS6 was originally detected in HONE-1 through the use of 10 μM cisplatin in culture medium. HONE1-CIS6 could represent a cisplatin-resistant cell subtype of HONE-1; such subtypes are more aggressive and are associated with higher rates of disease recurrence. *RANBP1*, *MCM5*, *EIF3B*, *PSMA6*, *NPM1*, and *HSP90AB1* were identified through RT-PCR analysis of HONE-1 and HONE1-CIS6 as the genes most significantly involved in the MYC_targets_v1 regulatory pathway in the seventh and ninth cell subtypes; moreover, *MCM5*, *RANBP1*, and *HSP90AB1* were significantly overexpressed in HONE1-CIS6.

## 4. Materials and Methods

This research was approved by the Institutional Review Board of National Cheng Kung University Hospital (IRB No: B-ER-108-424) and Institutional Animal Care and Use Committee of National Cheng Kung University (IACUC Approval No: 109137) and complied with medical research protocols established in the Declaration of Helsinki by the World Medical Association.

### 4.1. Establishment of an Experimental Animal Model

Male C57BL/6J mice raised on the Tainan campus of the National Institutes of Health, Taiwan, were used in experiments in this study in compliance with the guidelines of the Laboratory Animal Care and Use Committee of National Cheng Kung University. Mice were randomly divided into a control group and experimental group: the control group received normal drinking water, and the experimental group received drinking water with 4-NQO (200 μg/mL) (SIGMA-ALDRICH, N8141-5G) and arecoline (200 μg/mL) (TCI, A0523). All mice in the experimental group, except for those sacrificed at week 8, were treated for 16 weeks; then, they were either sacrificed or given normal drinking water thereafter until being sacrificed at weeks 20 or 29. Observation and analysis confirmed that the treatment stimulated healthy oral mucosa cells in mice to evolve into precancerous lesions and then into cancerous and malignant tissues.

### 4.2. Tongue Cell Isolation

After mice were sacrificed through carbon dioxide asphyxiation, incisional biopsy was performed on lesions on their tongues, and the specimens obtained were fixed in 10% buffered formalin. The specimens were stained with hematoxylin and eosin (H&E) for histopathological examination, and whole lesions were excised for subsequent scRNA-seq analysis. Tongue lesions were rinsed with 1× phosphate-buffered saline (PBS) (VWR, 97062-730), cut with a scalpel and soaked in gentleMACS™ C tubes (MACS, 5190703020) containing cell culture medium (DMEM/F12; Simply, CC131-1000) and tissue dissociation enzyme (neutral protease Dispase II (100 U/MG; SIGMA, D4693-1G) and Collagenase IV (0.1 mg/mL; SIGMA, C5138-100 MG)). Then, they were placed in an automatic tissue homogenization/single-cell separator (gentleMACSTM Octo Dissociator with Heaters) and maintained at 37 °C for 10 min to separate tissues into single cells. Subsequently, the lesions were placed in an incubator and maintained at 5% CO_2_ and a temperature of 37 °C for 3 min and then filtered through a 40-μm cell strainer (Falcon, 352340). The cell-containing suspension was collected, and 1× red blood cell lysis buffer (10× red blood cell lysis buffer; BD, 555899) was added. After lysis of red blood cells, the suspension was centrifuged at 1500 rpm for 5 min to remove the supernatant, washed with PBS containing 0.04% bovine serum albumin (Cyrusbioscience, 101-9048-46-8), and then centrifuged in PBS. Next, 10 μL of the final cell suspension was mixed with 10 μL of trypan blue (VWR, 72-57-1), and an automatic cell counter was used to determine the number of cells. Finally, the concentration was adjusted to 1000 cells/μL for microfluidic cell sorting.

10× Genomics Platform and 3′ Gene Expression Library Construction [47] were performed follow the manufactural recommendation protocols and list in Supplementary Materials Table S1.

### 4.3. Next-Generation Sequencing

The 3′ gene expression library, constructed as described in the previous section, was subjected to next-generation sequencing with the Illumina NextSeq 500/550 High-Output v2.5 Kit (150 cycles) (20024907) and sample indices. The parameters for paired-end, single indexing sequencing were as follows: Read 1, 28 cycles; i7 Index, 8 cycles; Read 2, 91 cycles; and Library Loading NextSeq 500/550, 1.5 pM [48].

### 4.4. Bioinformatic Analysis of Next-Generation Sequencing Data

After next-generation sequencing, Cellranger (v3.1.0, 10x Genomics) was used to analyze sequencing reads [49] and perform comparisons with original data for the mouse reference genome (mm10) by using STAR (RRID:SCR_005622) [50]—where “Count” is used for reads alignment, filtering, barcode counting, ΜMI counting, cell clustering, and gene expression analysis with the cell barcode and “aggr” is used to aggregate sample results [51]—and output the cloupe file to the Loupe Cell Browser (10x Genomics, v.3.1.0) [52].

### 4.5. Cell Line

The cell lines of the human nasopharyngeal carcinoma cell line (HONE-1) and drug-resistant human nasopharyngeal carcinoma cell line (HONE1-CIS6) screened using 10 μM cisplatin (Hospira Australia Pty Ltd.) were employed in the present study [53]. These two cell lines were provided by the Laboratory of Distinguished Professor Dar-Bin Shieh, Institute of Oral Medicine, National Cheng Kung University, Tainan, Taiwan. The cell lines were cultured directly from liquid nitrogen storage cells that were validated on 12/10/2013 (Supplementary Materials Figures S5 and S6). The cell lines were cultured with an RPMI 1640 cell culture medium (Gibco^TM^, 31800-014) containing 2 mM L-glutamine (Simply, CC515-0100) with the addition of 2 g/L sodium bicarbonate (Millipore, 144-55-8), 10% fetal bovine serum (Gibco, A38401), 1% L-glutamine (Simply, CC515-0100), and 1% antibiotic/antimycotic (Simply, CC501-0100). For the drug-resistant human nasopharyngeal carcinoma cell line (HONE1-CIS6), 10 μM cisplatin was added to the cell culture medium to preserve the cell characteristics.

RNA Expression Assay details were listed in Supplementary Materials Table S3. Protein Expression Assay details were listed in Supplementary Materials Figure S4.

## 5. Conclusions

In conclusion, a mouse model of oral tongue cell carcinogenesis induced by 4NQO and areocline administration enabled the transition of cells from healthy to precancerous and finally to cancerous to be studied with minimal individual heterogeneity. Tongue lesions obtained from the 16- and 29-week experimental groups were analyzed through scRNA-seq and compared with those obtained from the control group; 17 cell subtypes involved in carcinogenesis were identified, and the seventh (stem cell) and ninth (keratinocyte) oncogenic subtypes had the most significant involvement in the pathway related to *MYC*. The results were further validated by the presence of the cisplatin-resistant cell line HONE1-CIS6 with significant overexpression of *MCM5*, *RANBP1*, and *HSP90AB1*. These cell subtype biomarkers in oral cancer carcinogenesis can be applied in the detection of patients with precancerous lesions in the identification of high-risk populations and as a treatment target.

## Figures and Tables

**Table 1:** Next-generation sequencing mapping results of the mouse genome (mm10) in the control and experimental groups.

*Type*	Mean Reads per Cell	Reads Mapped to Genome
***16W_CONTROL_1***	3,779,830	95.9%
***16W_CONTROL_2***	120,426	94.3%
***16W_CONTROL_3***	71,885	93.4%
***16W_TREAT_1***	5,963,561	95.9%
***16W_TREAT_2***	205,687	92.6%
***16W_TREAT_3***	112,034	92.7%
***29W_CONTROL_1***	2,995,150	98.4%
***29W_CONTROL_2***	32,436	94.7%
***29W_TREAT_1***	1,335,636	97.7%
***29W_TREAT_2***	23,521	92.6%
***29W_TREAT_3***	14,880	92.9%

**Table 2:** Cell number of each of the 17 cell subtypes and their proportion in relation to the total cell composition in the four groups (16- and 29-week control and experimental groups).

Cell Number	2271	2022	1069	847	749	737	401	399	351	303	276	262	254	214	72	68	39
Subtype	0	1	2	3	4	5	6	7	8	9	10	11	12	13	14	15	16
16W_CTRL	192	239	521	440	109	325	2	98	38	10	5	201	6	14	7	12	9
16W_TREAT	118	82	354	340	47	332	1	16	26	9	5	29	7	5	3	8	24
29W_CTRL	224	1	85	27	31	33	173	1	11	10	33	0	26	0	2	18	1
29W_TREAT	1717	1700	109	40	562	47	225	284	276	274	233	32	215	195	60	30	5
**Within Cluster**																	
16W_CTRL	8%	12%	49%	52%	15%	44%	0%	25%	11%	3%	2%	77%	2%	7%	10%	18%	23%
16W_TREAT	5%	4%	33%	40%	6%	45%	0%	4%	7%	3%	2%	11%	3%	2%	4%	12%	62%
29W_CTRL	11%	0%	8%	3%	4%	4%	43%	0%	3%	3%	12%	0%	10%	0%	3%	26%	3%
29W_TREAT	76%	84%	10%	5%	75%	6%	56%	71%	79%	90%	84%	12%	85%	91%	83%	44%	13%
**Between Cluster**																	
16W_CTRL	9%	11%	23%	20%	5%	15%	0%	4%	2%	0%	0%	9%	0%	1%	0%	1%	0%
16W_TREAT	8%	6%	25%	24%	3%	24%	0%	1%	2%	1%	0%	2%	0%	0%	0%	1%	2%
29W_CTRL	35%	0%	12%	4%	4%	5%	25%	0%	2%	1%	5%	0%	4%	0%	0%	3%	0%
29W_TREAT	29%	28%	2%	1%	9%	1%	4%	5%	5%	5%	4%	1%	4%	3%	1%	0%	0%

## Supplementary Materials

The following are available online at https://www.mdpi.com/1422-0067/21/21/8171/s1.
