# Supplementary material for: Single-Cell Analysis of Different Stages of Oral Cancer Carcinogenesis in a Mouse Model
Source: Int J Mol Sci. 2020 Oct 31;21(21):8171. doi: 10.3390/ijms21218171 (PMC7662772; doi:10.3390/ijms21218171)
Supplement: Supplementary file 1 [file ijms-21-08171-s001.pdf]

Supplement

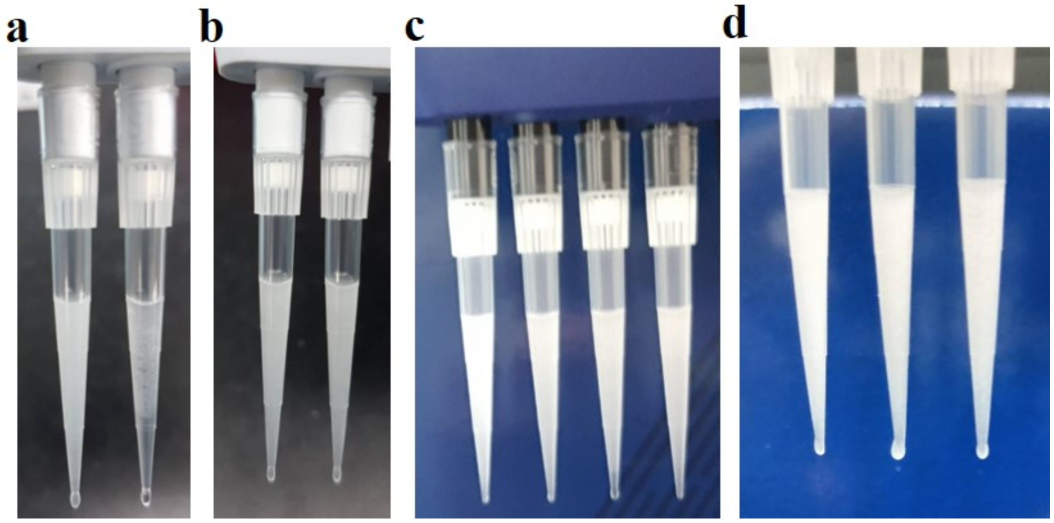

Supplemental Figure 1. Gel beads in emulsions (GEMs) generated by a single cell encapsulated in oil droplets. (**a**) After 29 weeks, cells in the experimental group (left) and control group (right) were encapsulated to produce GEMs; the control group exhibited a turbid, uneven distribution. (**b**) GEMs generated by repackaging cells obtained from the experimental and control groups at 29 weeks exhibited a uniform distribution. (**c**) The second batch of cells obtained from the experimental and control groups at 16 weeks generated GEMs after wrapping. (**d**) The second batch of cells obtained from the experimental and control groups at 29 weeks produced GEMs after wrapping.

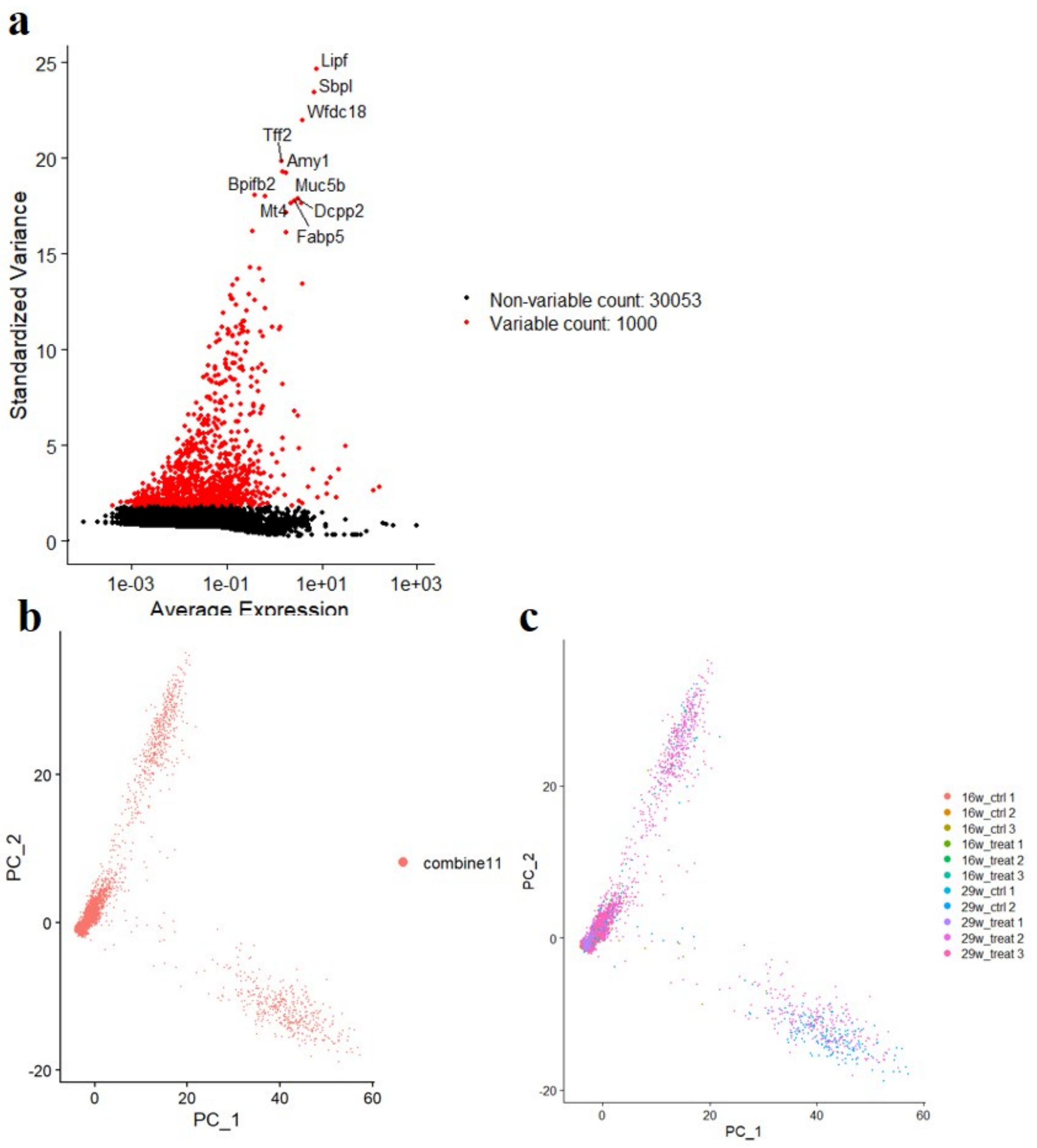

Supplemental Figure 2. Calibration and linear dimensionality reduction data. (**a**) Variable feature plot displaying the expression levels of the 31 053 genes in all cells; the 1000 genes with the highest expression levels were selected for subsequent analysis, and the top 10 genes are labeled. (**c**) Principal component analysis was performed to determine the positions of 11 samples, which are indicated by a different color for each of the 11 samples.

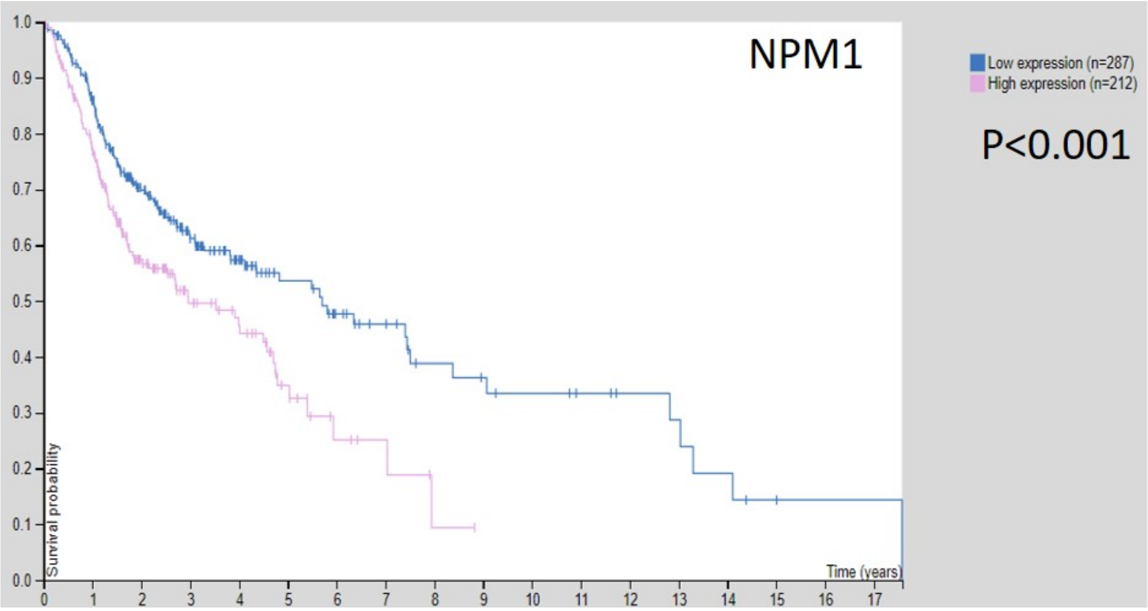

Supplemental Figure 3. Effect of NPM1 expression on the survival of patients with head and neck cancer. Data from The Cancer Genome Atlas database was analyzed to determine the relationship between NPM1 mRNA expression and the survival rate of patients with head and neck cancer. The number of patient specimens with a high expression of *NPM1* mRNA was 212. The number of patient specimens with low expression of *NPM1* mRNA was 287. A high expression of NPM1 was correlated with a lower likelihood of survival.

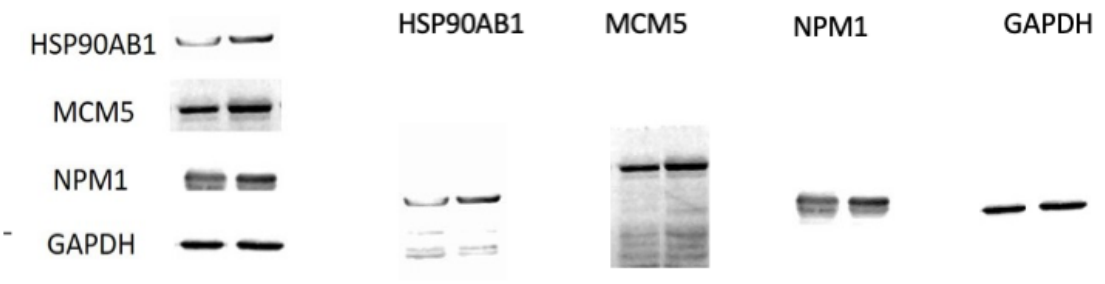

Supplemental Figure 4. The original PVDF membrane images of the Western Blotting of HSP90AB1, MCM5, NPM1, and GAPDH.

Protein extraction: the mouse tongues were cut with scissors and placed in a 1.5-ml microcentrifuge tube containing 2 μl of protease inhibitor (Geneaid) and 198 μl of Cytobuster Protein Extraction Reagent (Novagen, 71009-4). The tissues were ground using a micropestle, maintained at room temperature for 5 min and then centrifuged at 15 000 *g* for 5 min at 4°C. After centrifugation, the supernatant was transferred to a new microcentrifuge tube. With the protein extracted, the protein concentration was calculated so that protein samples could be prepared. To prepare these samples, 50 μg of protein was combined with 1 μl of reducing buffer (Lonza, 00193861B), 5 μl of loading dye (Protein Aaasy Dye Reagent Concentrate, Bio-Rad, 500-0006) and a sufficient amount of water to yield a volume of 20 μl; this mixture was then placed in a dry bath at a temperature of 100°C for 5 min. The protein samples were separated through sodium dodecyl sulfate polyacrylamide gel electrophoresis and then transferred to a polyvinylidene difluoride (PVDF; Millipore) membrane. The membrane was then placed in Tris-buffered saline combined with Tween 20 (TBST) and shaken on a flat rotary shaker for 1 h. The TBST solution was used to wash the PVDF membrane three times, with shaking at 50 rpm for 5 min each time. After the addition of the primary antibody, the solution was shaken at 50 rpm overnight on a flat rotary shaker at a temperature of 4°C (Supplemental Table 4). The next day, the solution was removed, and the PVDF membrane was washed three times with TBST, with shaking at 50 rpm for 5 min each time. Next, the secondary antibody was added, and the solution was shaken at 50 rpm on a flat rotary shaker for 1 h at room temperature. The PVDF membrane was washed with TBST three times, with shaking at 50 rpm for 5 min each time. Finally, Immobilon TM Western Chemiluminescent HRP Substrate (Millipore, P90720) was prepared at a ratio of 1:1. The appropriate amount was dropped onto the PVDF membrane to achieve coloration. Images were captured using a luminescence fluorescence image capture analyzer, and protein expression was quantified using Image J software.

Supplement Figure 5.

Cell Line DNA Typing Report

Mission Biotech
10F-3, No.3, Yuanchi Street
Nangang, Taipei
Taiwan 115
Tel: 886 2 26557128
Email: service@missionbio.com.tw

Case Number:CID20130174
Report Date:12/19/2013

Sample Information：

i. Applicant Name:謝達斌 Dar-BinShieh
ii. Institution:成功大學醫學院口腔醫學研究所 Institute of Oral Medicineand Department of Stomatology, National Cheng Kung University, Tainan, Taiwan
iii. Sample Description:HONE1
iv. Sample type: Cell Pellet
v. Sample Received Date:12/10/2013

Allele table for the tested cell DNA

STR Locus	Repeat Numbers
D5S818	11,12
D13S317	10,12,13.3
D7S820	10
D16S539	9,10,11
vWA	14,16
TH01	7,9
Amelogenin	X
TPOX	8,12
CSF1PO	10,11
D21S11	27,30

Test Description：

CaseNumber: CID20130174
Test Date: 12/12/2013

Sample was extracted by Roche MagNA Pure Compact System.
DNA conc.= 109.9 ng/μl
OD260/280 = 2.08
OD260/230 = 2.04

This test was performed by using the PromegaGenePrint® 10 System and analyzed by ABI PRISM 3730 GENETIC ANALYZER and GeneMapper® Software V3.7.

Verified by:
Laboratory Director(Title)

Liang Kuei Chang

12/16/2013

Allele Report

Case Number: CID20130174

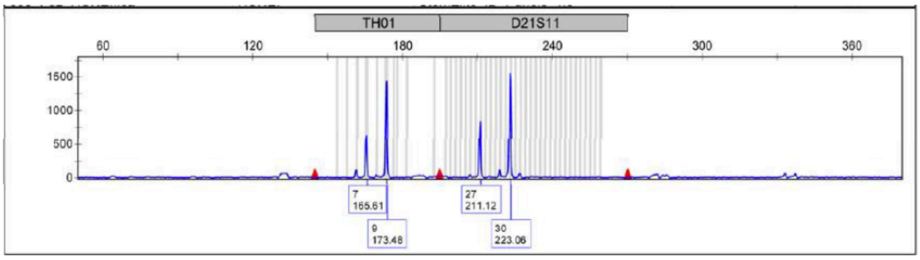

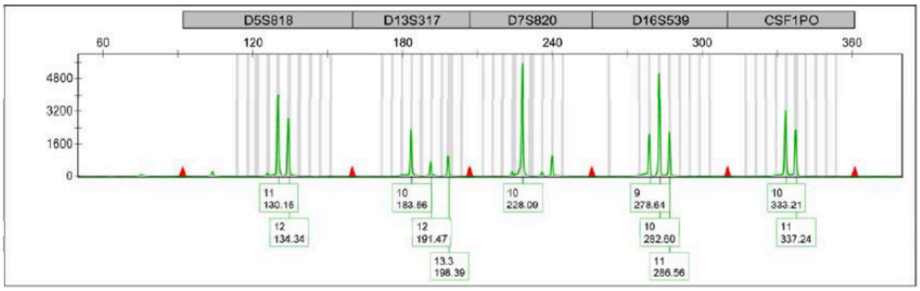

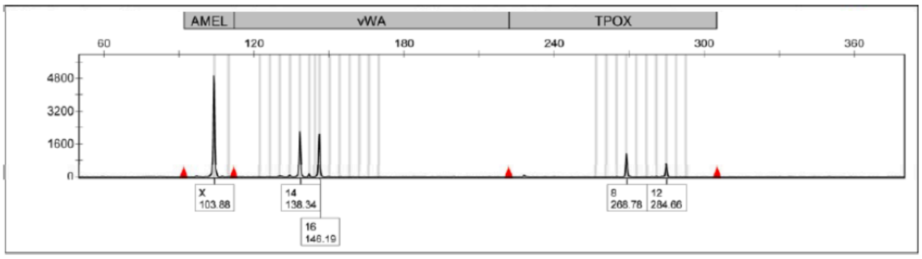

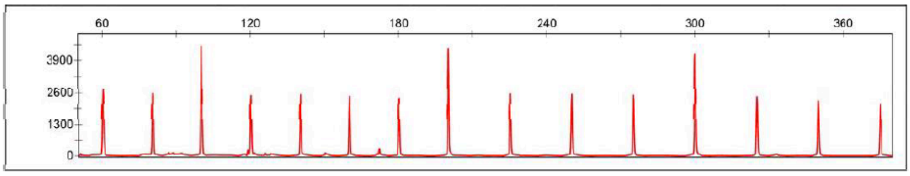

	Dye	Sample File Name	Marker	Allele	Size	Height	Area
1	B,1	003_F02_HONE1.fsa	TH01	7	165.61	643	3065
2	B,2	003_F02_HONE1.fsa	TH01	9	173.48	1436	6638
3	B,3	003_F02_HONE1.fsa	D21S11	27	211.12	839	3967
4	B,4	003_F02_HONE1.fsa	D21S11	30	223.06	1561	7405
5	G,1	003_F02_HONE1.fsa	D5S818	11	130.15	4036	18612
6	G,2	003_F02_HONE1.fsa	D5S818	12	134.34	2883	13131
7	G,3	003_F02_HONE1.fsa	D13S317	10	183.56	2345	11125
8	G,4	003_F02_HONE1.fsa	D13S317	12	191.47	724	3490
9	G,5	003_F02_HONE1.fsa	D13S317	13.3	198.39	1055	4936
10	G,6	003_F02_HONE1.fsa	D7S820	10	228.09	5574	28551
11	G,7	003_F02_HONE1.fsa	D16S539	9	278.64	2075	10531
12	G,8	003_F02_HONE1.fsa	D16S539	10	282.6	5044	25460
13	G,9	003_F02_HONE1.fsa	D16S539	11	286.56	2183	11131
14	G,10	003_F02_HONE1.fsa	CSF1PO	10	333.21	3221	17027
15	G,11	003_F02_HONE1.fsa	CSF1PO	11	337.24	2337	12476
16	Y,1	003_F02_HONE1.fsa	AMEL	X	103.88	4944	23088
17	Y,2	003_F02_HONE1.fsa	vWA	14	138.34	2205	10390
18	Y,3	003_F02_HONE1.fsa	vWA	16	146.19	2085	9818
19	Y,4	003_F02_HONE1.fsa	TPOX	8	268.78	1132	5573
20	Y,5	003_F02_HONE1.fsa	TPOX	12	284.66	622	3233

Allelic Ladder

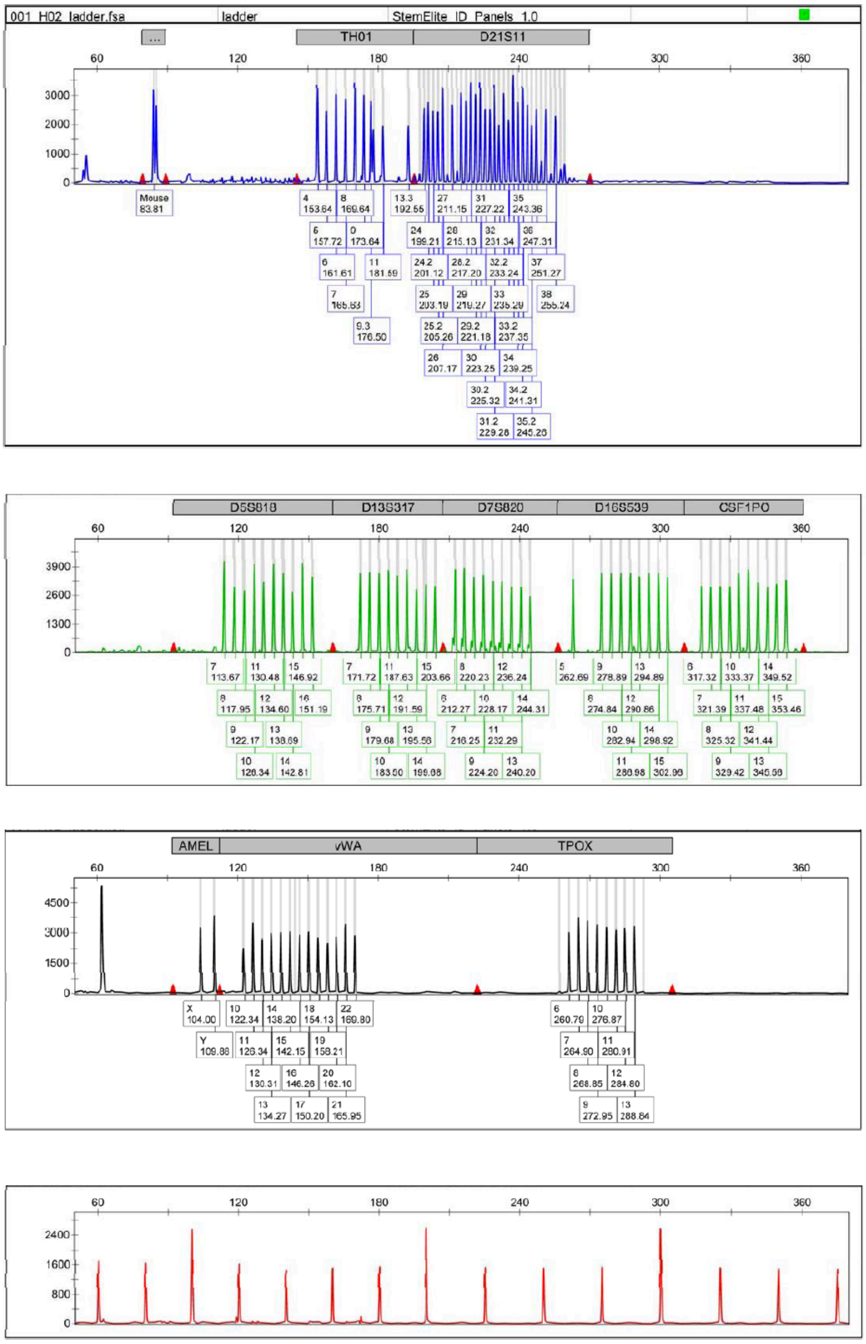

- *This STR analysis testing service is for research purposes only, andis not to be used for clinical diagnosis or applications involving humans.*
- *This test is just for typing the specific loci of the applied sample. The applicants may have to compare the results with the database of some bioresource institutes such as ATCC, JCRB or DSMZ by themselves.*

Genelabs Life science www.genelabs.com.tw
Phone:(02)26557678, (04)22633813, (06)2094380
Freecall:0800-231914,0800-094380

Supplement Figure 6.

Cell Line DNA Typing Report

Mission Biotech
10F-3, No.3, Yuanchi Street
Nangang, Taipei
Taiwan 115
Tel: 886 2 26557128
Email: service@missionbio.com.tw

Case Number:CID20130175
Report Date:12/19/2013

Sample Information:

i. Applicant Name:謝達斌 Dar-BinShieh
ii. Institution:成功大學醫學院口腔醫學研究所 Institute of Oral Medicineand Department of Stomatology, National Cheng Kung University, Tainan, Taiwan
iii. Sample Description:HONE1-CIS6
iv. Sample type: Cell Pellet
v. Sample Received Date:12/10/2013

Allele table for the tested cell DNA

STR Locus	Repeat Numbers
D5S818	12
D13S317	10
D7S820	10,12
D16S539	9,10
vWA	14,16
TH01	6,7,9
Amelogenin	X
TPOX	8,12
CSF1PO	10,11
D21S11	27,30

Test Description:

CaseNumber: CID20130175
Test Date: 12/12/2013

Sample was extracted by Roche MagNA Pure Compact System.
DNA conc.= 125.0 ng/μl
OD260/280 = 2.08
OD260/230 = 2.12

This test was performed by using the PromegaGenePrint® 10 System and analyzed by ABI PRISM 3730 GENETIC ANALYZER and GeneMapper® Software V3.7.

Verified by:
Laboratory Director(Title)

Liang Kuei Chang

12/16/2013

Allele Report

Case Number: CID20130175

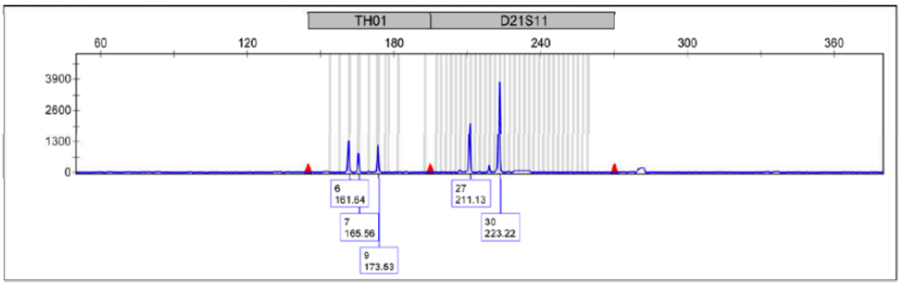

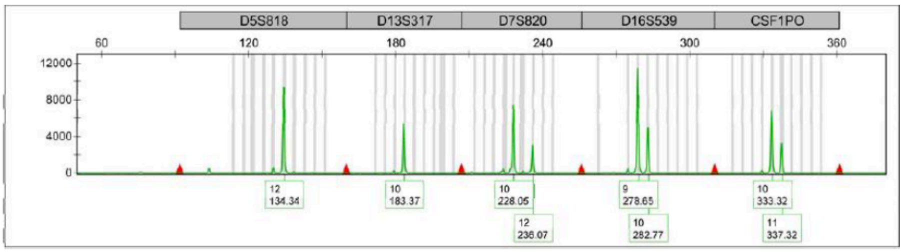

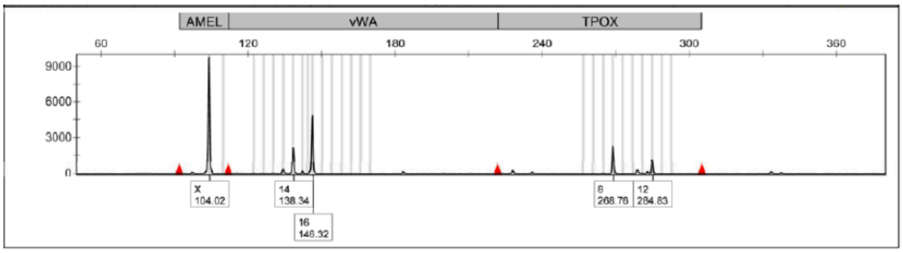

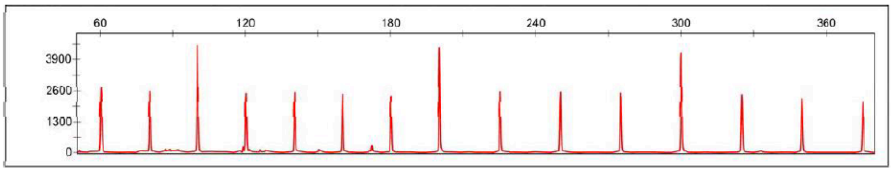

	Dye	Sample File Name	Marker	Allele	Size	Height	Area
1	B,1	002_G02_HONE1-CIS6.fsa	TH01	6	161.64	1329	6112
2	B,2	002_G02_HONE1-CIS6.fsa	TH01	7	165.56	838	3681
3	B,3	002_G02_HONE1-CIS6.fsa	TH01	9	173.53	1171	5308
4	B,4	002_G02_HONE1-CIS6.fsa	D21S11	27	211.13	2075	9523
5	B,5	002_G02_HONE1-CIS6.fsa	D21S11	30	223.22	3787	17879
6	G,1	002_G02_HONE1-CIS6.fsa	D5S818	12	134.34	9331	43156
7	G,2	002_G02_HONE1-CIS6.fsa	D13S317	10	183.37	5408	25163
8	G,3	002_G02_HONE1-CIS6.fsa	D7S820	10	228.05	7498	36972
9	G,4	002_G02_HONE1-CIS6.fsa	D7S820	12	236.07	3087	15200
10	G,5	002_G02_HONE1-CIS6.fsa	D16S539	9	278.65	11493	57548
11	G,6	002_G02_HONE1-CIS6.fsa	D16S539	10	282.77	5021	25546
12	G,7	002_G02_HONE1-CIS6.fsa	CSF1PO	10	333.32	6806	34986
13	G,8	002_G02_HONE1-CIS6.fsa	CSF1PO	11	337.32	3270	16528
14	Y,1	002_G02_HONE1-CIS6.fsa	AMEL	X	104.02	9763	44820
15	Y,2	002_G02_HONE1-CIS6.fsa	vWA	14	138.34	2155	10390
16	Y,3	002_G02_HONE1-CIS6.fsa	vWA	16	146.32	4909	23029
17	Y,4	002_G02_HONE1-CIS6.fsa	TPOX	8	268.76	2347	11189
18	Y,5	002_G02_HONE1-CIS6.fsa	TPOX	12	284.83	1133	5511

Allelic Ladder

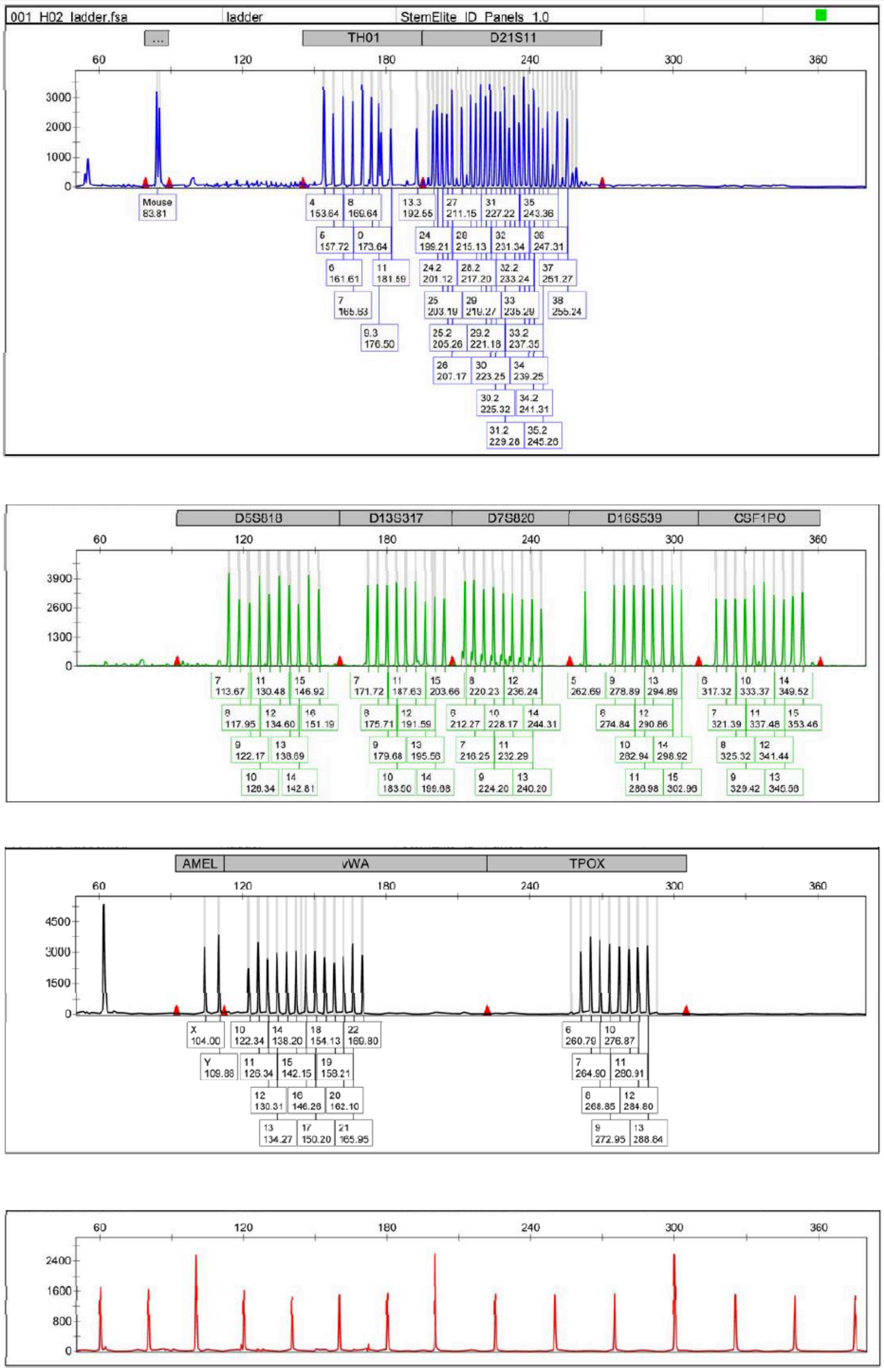

- *This STR analysis testing service is for research purposes only, andis not to be used for clinical diagnosis or applications involving humans.*
- *This test is just for typing the specific loci of the applied sample. The applicants may have to compare the results with the database of some bioresource institutes such as ATCC, JCRB or DSMZ by themselves.*

Genelabs Life science www.genelabs.com.tw
Phone:(02)26557678, (04)22633813, (06)2094380
Freecall:0800-231914,0800-094380

Supplemental Table 1.

Protocol for Generating Single-Cell 3' Gene Expression Libraries from Single Cells.

Steps	Procedures	Time	Store
**Cell Preparation**			
Cell Dissociation		1.5 h	
**Step 1 – GEM Generation & Barcoding**			
1-1	Prepare Reaction Mix	20 min	
1-2	Load Chromium Next GEM Chip G	10 min	
1-3	Run the Chromium Controller	18 min	
1-4	Transfer GEMs	3 min	
1-5	GEM-RT Incubation	55 min	4°C ≤72 h or −20°C ≤1 week
**Step 2 – Post GEM-RT Cleanup & cDNA Amplification**			
2-1	Post GEM RT-Cleanup – Dynabead	45 min	
2-2	cDNA Amplification	40 min	4°C ≤72 h or −20°C ≤1 week
2-3	cDNA Cleanup – SPRIselect	20 min	4°C ≤72 h −20°C ≤4 weeks
2-4	cDNA QC & Quantification	50 min	
**Step 3 – 3' Gene Expression Library Construction**			
3-1	Fragmentation, End Repair & A-tailing	50 min	
3-2	Post Fragmentation, End Repair & A-tailing Double Sided Size Selection – SPRIselect	30 min	
3-3	Adaptor Ligation	25 min	
3-4	Post Ligation Cleanup- SPRIselect	20 min	
3-5	Sample Index PCR	40 min	4°C ≤72 h
3-6	Post Sample Index PCR Double Sided Size Selection – SPRIselect	30 min	4°C ≤72 h or -20°C long term
3-7	Post Library Construction QC	50 min	

First, 50% glycerol (JT Baker, 2136-01) was added to the Chromium Next GEM Chip G (Chip G; PN-1000120). Next, an appropriate amount of nuclease-free water (Thermo Fisher Scientific, R0581) and the corresponding amount of the cell suspension were added to the master mix [18.8 μl of RT Reagent B (PN2000165), 2.4 μl of Template Switch Oligo (PN3000228), 2.0 μl of Reducing Agent B (PN2000087) and 8.7 μl of RT Enzyme C (PN2000085)] to achieve a total volume of 75 μl. Subsequently, 70 μl of the combined master mix and cell suspension was added to the first row of Chip G (PN2000177). After single-cell 3' v3.1 gel beads (PN2000164) had been shaken for 30 s, 50 μl was extracted and added to the second row of Chip G, and 45 μl of partitioning oil (PN2000190) was added to the third row of Chip G. The 10X Gasket (PN370017) was then connected to the Chromium Controller and activated to coat the oil droplets. After processing, the gasket was removed and the chip holder was opened to form a 45° well. The volume of the first and second rows of Chip G was assessed, and then, 100 μl of gel beads in emulsion (GEMs) were transferred from the third row of Chip G to 8-row polymerase chain reaction (PCR) tubes. The GEMs then underwent incubation at a temperature of 53°C for 45 min and 85°C for 5 min before being maintained at 4°C.

To form Dynabeads, 125 μl of Recovery Agent (PN220016) was added to each sample, and the samples were kept at room temperature for 2 min. Next, 125 μl of the combined Recovery Agent and partitioning oil was extracted from the bottom of the tube, and a Dynabeads cleanup mix was prepared [182 μl of Cleanup Buffer (PN2000088), 8 μl of Dynabeads MyOne SILANE (PN2000048), 5 μl of Reducing Agent B (PN2000087), and 5 μl of nuclease-free water (Thermo Fisher Scientific, R0581)]. Each sample was supplemented with 200 μl of the cleanup mix and maintained at room temperature for 10 min. The samples were then placed in the 10X Magnetic Separator (PN120250) in the high position (10X Magnetic Separator-High) until the liquid was clear and then removed. Next, 300 μl of 80% alcohol was added to each sample. After 30 s, the alcohol was removed and 200 μl of

80% alcohol was added to each sample. After 30 s, the alcohol was removed again, and then, the sample was removed from the magnetic holder and centrifuged and placed in the 10X Magnetic Separator in the low position (10X Magnetic Separator-Low) to remove alcohol and air dry for 1 min. The samples were removed from the magnetic base, immediately supplemented with 35.5 µl of Elution Solution I [98 µl of elastic buffer (EB) (Qiagen, 19086), 1 µl of 10% Tween 20 (Sigma, P9416-50ML) and 1 µl of Reducing Agent B (PN2000087)], and then maintained at room temperature for 2 min. They were then returned to the magnetic base in the 10X Magnetic Separator-Low until the liquid was clear, and 35 µl of the samples were transferred to a new 8-row PCR tube.

After preparation of a cDNA amplification mix [50 µl of Amp Mix (PN2000047) and 15 µl of cDNA primers (PN2000089)], 35 µl of it was added to each sample (35 µl). A reaction was induced as follows: the temperature was set at 98°C for 3 min, 98°C for 15 sec, 63°C for 20 sec and 72°C for 1 min for 1 cycle and then 63°C for 20 sec and 72°C for 1 min for 11 cycles before being set at 72°C for 1 min and then maintained at 4°C. cDNA Cleanup-SPRIselect with shaking the SPRIselect reagent (Beckman Coulter, B23318); 60 µl (0.6X) was added to each sample, and then, the samples were maintained at room temperature for 5 min before being placed in the 10X Magnetic Separator-High until the liquid was clear. The supernatant was removed, and 200 µl of 80% alcohol was added to each sample. After 30 s, the alcohol was removed, and 200 µl of 80% alcohol was added to each sample. After 30 s, the alcohol was again removed. The sample was then removed from the magnetic stand, centrifuged and placed in the 10X Magnetic Separator-Low to remove the remaining alcohol and air dry for 2 min. The sample was then removed from the magnetic base, supplemented with 40.5 µl of EB (Qiagen,19086) and maintained at room temperature for 2 min. Finally, 40 µl of the sample was transferred to a new 8-row PCR tube for measurement of the cDNA concentration. Fragmentation, end repair, and A-tailing were performed with a fragmentation mix [5 µl of Fragmentation Buffer (PN2000091) and 10 µl of Fragmentation Enzyme (PN2000090)] on ice. After 10 µl of cDNA was placed in a new 8-row PCR tube, 25 µl of EB (Qiagen, 19086) was added to each sample, and then, 15 µl of the fragmentation mix was added to each sample. The sample was placed in a thermal cycler under the following conditions: precool block at 4°C, fragmentation at 32°C for 5 min, end repair and A-tailing at 65°C for 30 min and finally maintenance at 4°C. Each sample was supplemented with 30 µl (0.6X) of shaken SPRIselect reagent and maintained at room temperature for 5 min before being placed in the 10X Magnetic Separator-High until the liquid was clear. After 75 µl of the supernatant had been placed in a new 8-row PCR tube, each sample was supplemented with 10 µl (0.8X) of shaken SPRIselect reagent (Beckman Coulter, B23318), maintained at room temperature for 5 min and then placed in the 10X Magnetic Separator-High until the liquid was clear. Next, 80 µl of the supernatant was removed, and 125 µl of 80% alcohol was added to each sample. After 30 s, the alcohol was removed and replaced with another 125 µl of 80% alcohol. After 30 s, the alcohol was again removed; samples were removed from the magnetic stand and then centrifuged and placed in the 10X Magnetic Separator-Low until the liquid was clear. The remaining alcohol was then removed, and samples were removed from the magnetic stand and centrifuged, supplemented with 50.5 µl of EB (Qiagen,19086) and maintained at room temperature for 2 min. Next, samples were placed in the 10X Magnetic Separator-Low until the liquid was clear, and 50 µl of the samples were placed in a new 8-row PCR tube.

Adaptor ligation was performed using an adaptor ligation mix [20 µl of Ligation Buffer (PN2000092), 10 µl of DNA Ligase (PN220110) and 20 µl of Adaptor Oligos (PN2000094)]. After the addition of 50 µl of the adaptor ligation mix to each 50-µl sample, a temperature reaction was induced as follows: the temperature was set at 20°C 15 min and then maintained at 4°C. Each sample was supplemented with 80 µl (0.8X) of shaken SPRIselect reagent and maintained at room temperature for 5 min before being placed in the 10X Magnetic Separator-High until the liquid was clear. The supernatant was removed, and 200 µl of 80% alcohol was added to each sample. After 30 s, the alcohol was removed and replaced with 200 µl of 80% alcohol. After 30 s, the alcohol was again removed. Samples were taken out of the magnetic holder, centrifuged and placed in the 10X Magnetic Separator-Low. The remaining alcohol was removed, and the samples were air dried. After 2 min, the samples were removed from the magnetic holder and centrifuged. After being supplemented with 30.5 µl of EB, the samples were maintained at room temperature for 2 min and then placed in the 10X Magnetic Separator-Low until the liquid was clear. Next, 30 µl of the samples were placed in new 8-row PCR tubes.

A sample index PCR mix [50 µl of Amp Mix (PN2000047) and 10 µl of SI Primer (PN2000095)] (Supplemental Table 2) was prepared and used to perform sample index PCR. Each 30-µl sample was supplemented with 60 µl of the sample index PCR mix and 10 µl of the individual Chromiµm i7 Sample Index and then subjected to a temperature reaction under the following

conditions: temperature set at 98°C for 45 sec, 98°C for 20 sec, 54°C for 30 sec, 72°C for 20 sec and 98°C for 20 sec for 16 cycles and then set at 72°C for 1 min before being maintained at 4°C.

Post Sample Index PCR Double Sided Size Selection-SPRIselect Each sample was supplemented with 60 μl (0.6X) of shaken SPRIselect reagent and maintained at room temperature for 5 min. The samples were then placed in the 10X Magnetic Separator-High until the liquid was clear, and 150 μl of the supernatant was transferred to a new 8-row PCR tube. Each sample was supplemented with 20 μl of (0.8X) shaken SPRIselect reagent and maintained at room temperature for 5 min before being placed in the 10X Magnetic Separator-High until the liquid was clear; 165 μl of the supernatant was then removed, and 200 μl of 80% alcohol was added to each sample. After 30 s, the alcohol was removed, replaced with 200 μl of 80% alcohol and then removed again after 30 s. The samples were removed from the magnetic base and centrifuged, supplemented with 35.5 μl of EB and maintained at room temperature for 2 min before being placed in the 10X Magnetic Separator-Low until the liquid was clear. Finally, 35 μl of the samples was transferred to a new 8-row PCR tube for assessment of their concentration after establishment of a gene expression library.

Supplemental Table 2.

Sample indices of the NextSeq 500/550 High-Output v2.5 Kit (150 cycles) (20024907)

sample name	Index_Plate_Well	I7_Index_ID	index 1 sequence
TN-42_1	SI-GA-D11_1	SC0084	CTTTGCGG
TN-42_2	SI-GA-D11_2	SC0180	TGCACAAA
TN-42_3	SI-GA-D11_3	SC0276	AAGCAGTC
TN-42_4	SI-GA-D11_4	SC0372	GCAGTTCT
TN-43_1	SI-GA-C11_1	SC0083	GAGGATCT
TN-43_2	SI-GA-C11_2	SC0179	AGACCATA
TN-43_3	SI-GA-C11_3	SC0275	TCCTGCGC
TN-43_4	SI-GA-C11_4	SC0371	CTTATGAG
TN-44_1	SI-GA-B11_1	SC0082	GTTCCTCA
TN-44_2	SI-GA-B11_2	SC0178	AGGTACGC
TN-44_3	SI-GA-B11_3	SC0274	TAAGTATG
TN-44_4	SI-GA-B11_4	SC0370	CCCAGGAT

Supplemental Table 3.

Primers of the Real-Time Quantitative Polymerase Chain Reaction

PSMA6-RTF	GGTTTTGACCGCCACATTACC
PSMA6-RTR	GCCACCCTGGTTAATAGCCT
EIF3B-RTF	GGACCCGACCGACTTGAGA
EIF3B-RTR	TTGACCCGGAATGTGTGCTG
MCM5-RTF	AGCATTCGTAGCCTGAAGTCG
MCM5-RTR	CGGCACTGGATAGAGATGCG
RANBP1-RTF	CAAAACTGTTCCGATTTGCCTC
RANBP1-RTR	GGCGTGATGTAGTGGTTGG
HSP90AB1-RTF	CGAAGTTGGACAGTGGTAAAGAG
HSP90AB1-RTR	TGCCCAATCATGGAGATGTCT
NPM1-RTF	GCACTTAGTAGCTGTGGAGGA
NPM1-RTR	TTCACTGGCGCTTTTTCTTCA

RNA extraction: Mouse tongues were placed in a 1.5-ml microcentrifuge tube containing 1 ml of TRI reagent (Zymo, R2050-1-200). The tissues were cut with scissors, ground using a micropestle and then placed on ice for 5 min. After the addition of 200 µl of chloroform (PanReac AppliChem, A3691, 1000), it was shaken upside down to achieve an even distribution, placed on ice for 5 min and then centrifuged at 12 000 rpm at a temperature of 4°C for 15 min. The supernatant was transferred to a new 1.5-ml microcentrifuge tube and supplemented with an equal volume of isopropanol (PanReac AppliChem, A3928, 0500PE). The mixture was shaken upside down and mixed well. After resting on

ice for 10 min, it was then centrifuged at 12 000 rpm at a temperature of 4°C for 10 min to precipitate RNA. The supernatant was removed, leaving behind a white RNA pellet. After the addition of 1 ml of 70% alcohol (ECHO, 484000011107A-69EC) and a mixture of 5 diethyl pyrocarbonate (VWR, 71003-388) and water, the RNA pellet was washed and centrifuged at 7500 rpm at a temperature of 4°C for 5 min to remove the supernatant. The microcentrifuge tube was left open on a sterile operating table to air dry. After the alcohol had completely evaporated, 30 µl of diethyl pyrocarbonate water was added; RNA was purified, and its quality and concentration were assessed using a NanoDrop spectrophotometer before it was finally stored in a refrigerator at −80°C.

Reverse-transcription PCR (RT-PCR): in a PCR tube, 4 µl of 5X cDNA Synthesis Mix (PCR Biosystems, PB30.31-10) was combined with 1 µl of MltraScript 2.0 for cDNA synthesis (PCR Biosystems, PB30.31-10) and 2 µg of RNA with enough nuclease-free water (Thermo Fisher Scientific, R0581) to yield a total volume of 20 µl. The samples were placed in a PCR machine for a cDNA synthesis reaction with the temperature set at 55°C for 30 min and 95°C for 10 min before being maintained at 12°C. Subsequently, the cDNA underwent RT-PCR analysis in 8-row PCR tubes containing 10 µl of 2X qPCRBIO SyGreen Blue Mix (PCR Biosystems, PB20.16-01), 0.8 µl (10 µM) of forward primer, 0.8 µl (10 µM) of reverse primer, 1 µl of cDNA and 7.4 µl of nuclease-free water (Thermo Fisher Scientific, R0581) (Supplemental Table 3). The aforementioned q-PCR reaction mixture was placed in a real-time PCR apparatus, and real-time quantification was performed under the following conditions: temperature set at 95°C for 2 min, 95°C for 5 sec, 65°C for 30 sec and 95°C for 5 sec for 40 cycles and then 95°C for 15 sec, 60°C for 1 min and 95°C for 15 sec to melt the curve.

Supplemental Table 4.

Antibodies in the Western blot

Antibody	Brand	Dilution
Anti-BRK	Invitrogen	1:5000
Anti-GAPDH	proteintech	1:5000
Anti-MCM5	abcam	1:5000
Anti-NPM1	proteintech	1:3000
Anti-HSP90AB1	proteintech	1:2000
Goat anti-mouse IgG	Millipore	1:5000
Goat anti-rabbit IgG	Millipore	1:10000

Supplemental Table 5.

Significant Genes Expressed in Each of the 17 Cell Subtypes

cluster	order	gene	p_val	avg_logFC	pct.1	pct.2	p_val_adj
0	1	*Bpgm*	0.00E+00	1.3378	0.837	0.3	0.00E+00
0	2	*Fam46c*	0.00E+00	1.2019	0.786	0.244	0.00E+00
0	3	*Snca*	0.00E+00	1.1781	0.856	0.288	0.00E+00
0	4	*Mkrn1*	0.00E+00	1.1177	0.895	0.491	0.00E+00
0	5	*Epb41*	0.00E+00	1.0214	0.719	0.251	0.00E+00
0	6	*Hbb-bt*	0.00E+00	0.9622	0.995	0.489	0.00E+00
0	7	*Fam213a*	1.69E-279	0.9483	0.412	0.103	5.24E-275
0	8	*Hbb-bs*	0.00E+00	0.9466	0.998	0.709	0.00E+00
0	9	*Alas2*	0.00E+00	0.8486	0.934	0.351	0.00E+00
0	10	*Ube2o*	0.00E+00	0.7664	0.45	0.109	0.00E+00
0	11	*Fam220a*	0.00E+00	0.7386	0.716	0.282	0.00E+00
0	12	*Ube2l6*	0.00E+00	0.712	0.721	0.288	0.00E+00
0	14	*Isg20*	9.17E-252	0.656	0.625	0.252	2.85E-247
0	21	*Hba-a1*	9.07E-171	0.5559	0.996	0.53	2.82E-166
0	23	*Hba-a2*	1.45E-174	0.504	0.996	0.494	4.50E-170
0	30	*Car2*	1.84E-130	0.4005	0.357	0.13	5.72E-126
0	34	*Prdx2*	1.18E-166	0.3913	0.637	0.347	3.66E-162
0	39	*Gypa*	1.16E-80	0.3123	0.257	0.099	3.60E-76

cluster	order	gene	p_val	avg_logFC	pct.1	pct.2	p_val_adj
**1**	1	*Retnlg*	0.00E+00	4.4788	0.92	0.054	0.00E+00
**1**	2	*S100a9*	0.00E+00	3.3422	0.995	0.324	0.00E+00
**1**	3	*S100a8*	0.00E+00	3.341	0.996	0.312	0.00E+00
**1**	4	*Cxcl2*	0.00E+00	3.1766	0.656	0.057	0.00E+00
**1**	5	*Ifitm6*	0.00E+00	2.9945	0.876	0.078	0.00E+00
**1**	6	*Wfdc21*	0.00E+00	2.8492	0.94	0.067	0.00E+00
**1**	7	*Lcn2*	0.00E+00	2.795	0.95	0.12	0.00E+00
**1**	8	*Ngp*	0.00E+00	2.6969	0.811	0.081	0.00E+00
**1**	9	*Mmp9*	0.00E+00	2.6541	0.855	0.02	0.00E+00
**1**	10	*Ltf*	0.00E+00	2.6436	0.594	0.048	0.00E+00
**1**	13	*Camp*	0.00E+00	2.4163	0.705	0.117	0.00E+00
**1**	76	*Lyz2*	0.00E+00	1.4481	0.934	0.133	0.00E+00
**1**	84	*Chil3*	0.00E+00	1.3664	0.705	0.094	0.00E+00
**1**	141	*Hmgb2*	0.00E+00	1.1702	0.916	0.224	0.00E+00
**1**	145	*Serpinb1a*	0.00E+00	1.1458	0.519	0.081	0.00E+00
**1**	191	*Hmgn2*	0.00E+00	0.9585	0.568	0.102	0.00E+00
**1**	528	*H2afz*	1.83E-233	0.4555	0.691	0.276	5.67E-229

cluster	order	gene	p_val	avg_logFC	pct.1	pct.2	p_val_adj
**2**	1	*Hbb-bt*	0.00E+00	1.0692	0.996	0.555	0.00E+00

**2**	2	*Hba-a2*	0.00E+00	0.9751	0.997	0.559	0.00E+00
**2**	3	*Hba-a1*	0.00E+00	0.9109	0.998	0.59	0.00E+00
**2**	4	*Bpgm*	5.35E-240	0.8089	0.895	0.363	1.66E-235
**2**	5	*Hbb-bs*	1.50E-160	0.7467	0.998	0.746	4.66E-156
**2**	6	*Mkrn1*	4.61E-187	0.7158	0.918	0.541	1.43E-182
**2**	7	*Alas2*	2.77E-187	0.7046	0.937	0.426	8.60E-183
**2**	8	*Snca*	4.73E-199	0.7026	0.869	0.36	1.47E-194
**2**	9	*Ube2l6*	5.24E-177	0.6432	0.792	0.336	1.63E-172
**2**	10	*Isg20*	1.08E-164	0.6122	0.716	0.29	3.34E-160
**2**	12	*Fam220a*	9.87E-153	0.5884	0.758	0.334	3.07E-148
**2**	19	*Gypa*	1.59E-101	0.3229	0.359	0.108	4.95E-97
**2**	24	*Fam213a*	9.35E-49	0.2984	0.34	0.152	2.90E-44
**2**	25	*Isca1*	5.16E-55	0.2684	0.344	0.147	1.60E-50

**cluster**	**order**	**gene**	**p_val**	**avg_logFC**	**pct.1**	**pct.2**	**p_val_adj**
**3**	1	*Hba-a2*	0.00E+00	1.1189	1	0.569	0.00E+00
**3**	2	*Hba-a1*	0.00E+00	1.0922	1	0.6	0.00E+00
**3**	3	*Hbb-bs*	8.33E-195	0.773	1	0.752	2.59E-190
**3**	4	*Alas2*	3.84E-36	0.4687	0.702	0.459	1.19E-31

**3**	5	*Ube2l6*	4.80E-13	0.2763	0.481	0.375	1.49E-08
**3**	6	*Fam220a*	8.58E-12	0.2576	0.479	0.368	2.66E-07
**3**	7	*Hbb-bt*	1.32E-45	0.2535	0.996	0.565	4.10E-41

**cluster**	**order**	**gene**	**p_val**	**avg_logFC**	**pct.1**	**pct.2**	**p_val_adj**
**4**	1	*Apoe*	0.00E+00	3.0049	0.525	0.063	0.00E+00
**4**	2	*Cd74*	0.00E+00	2.4612	0.344	0.029	0.00E+00
**4**	3	*H2-Ab1*	8.43E-252	2.4507	0.286	0.025	2.62E-247
**4**	4	*H2-Eb1*	6.63E-238	2.4167	0.256	0.021	2.06E-233
**4**	5	*H2-Aa*	9.64E-278	2.3344	0.28	0.02	2.99E-273
**4**	6	*Ctss*	0.00E+00	2.2366	0.678	0.021	0.00E+00
**4**	7	*Lyz2*	5.98E-209	2.0643	0.71	0.257	1.86E-204
**4**	8	*S100a4*	0.00E+00	1.9809	0.618	0.056	0.00E+00
**4**	9	*Plac8*	2.61E-246	1.9717	0.549	0.132	8.11E-242
**4**	10	*Psap*	2.95E-303	1.94	0.706	0.205	9.15E-299
**4**	29	*Chil3*	2.30E-55	1.3452	0.425	0.197	7.15E-51

**4**	113	*H2afz*	4.75E-155	0.9992	0.754	0.326	1.48E-150
**4**	143	*Lgals1*	9.89E-263	0.9463	0.522	0.088	3.07E-258
**4**	233	*H2-K1*	7.74E-158	0.7733	0.646	0.215	2.40E-153

**cluster**	**order**	**gene**	**p_val**	**avg_logFC**	**pct.1**	**pct.2**	**p_val_adj**
**5**	1	*Hba-a2*	1.55E-271	1.0144	0.997	0.574	4.82E-267
**5**	2	*Hba-a1*	1.00E-262	0.9697	0.997	0.605	3.11E-258
**5**	3	*Hbb-bs*	3.68E-170	0.7575	0.997	0.755	1.14E-165
**5**	4	*Hbb-bt*	5.24E-94	0.5807	0.996	0.57	1.63E-89
**5**	5	*Alas2*	4.73E-65	0.5144	0.825	0.452	1.47E-60
**5**	6	*Fam220a*	1.20E-29	0.344	0.583	0.362	3.72E-25
**5**	7	*Ube2l6*	8.72E-26	0.3002	0.575	0.368	2.71E-21
**5**	8	*Isca1*	3.99E-16	0.2816	0.274	0.16	1.24E-11

cluster	order	gene	p_val	avg_logFC	pct.1	pct.2	p_val_adj
**6**	1	*Dcn*	0.00E+00	4.8504	1	0.027	0.00E+00
**6**	2	*Gsn*	0.00E+00	4.5078	1	0.19	0.00E+00
**6**	3	*Plpp3*	0.00E+00	3.1037	0.93	0.032	0.00E+00
**6**	4	*Igfbp4*	0.00E+00	3.0014	0.983	0.046	0.00E+00
**6**	5	*Col3a1*	0.00E+00	2.9549	0.875	0.007	0.00E+00
**6**	6	*Serping1*	0.00E+00	2.8792	0.978	0.024	0.00E+00
**6**	7	*Mfap5*	0.00E+00	2.8517	0.945	0.004	0.00E+00
**6**	8	*Col1a2*	0.00E+00	2.6834	0.95	0.004	0.00E+00
**6**	9	*Dpt*	0.00E+00	2.6559	0.92	0.003	0.00E+00
**6**	10	*Cdkn1c*	0.00E+00	2.6023	0.623	0.014	0.00E+00
**6**	12	*Igfbp5*	0.00E+00	2.5602	0.723	0.039	0.00E+00
**6**	31	*Sparc*	0.00E+00	2.2513	0.92	0.029	0.00E+00
**6**	51	*Igfbp7*	0.00E+00	1.9009	0.893	0.027	0.00E+00
**6**	68	*Mgp*	0.00E+00	1.7399	0.459	0.023	0.00E+00
**6**	69	*Ebf1*	0.00E+00	1.7373	0.845	0.043	0.00E+00
**6**	78	*Lgals1*	0.00E+00	1.6532	0.913	0.087	0.00E+00
**6**	90	*Sptbn1*	0.00E+00	1.5671	0.943	0.14	0.00E+00
**6**	281	*Timp3*	0.00E+00	0.9492	0.603	0.047	0.00E+00
**6**	370	*Psap*	4.50E-203	0.829	0.87	0.216	1.40E-198
**6**	466	*S100a4*	1.99E-114	0.7451	0.439	0.083	6.17E-110

**6**	541	*Hspb1*	1.40E-174	0.6929	0.449	0.061	4.36E-170
**6**	1043	*Nupr1*	3.27E-306	0.4709	0.701	0.097	1.02E-301
**6**	1180	*H2-K1*	2.78E-83	0.4197	0.686	0.228	8.64E-79
**6**	1237	*Apoe*	9.16E-61	0.4005	0.337	0.087	2.85E-56
**6**	1566	*Gng11*	5.64E-122	0.2874	0.367	0.055	1.75E-117
**6**	1582	*Cavin2*	2.70E-127	0.2771	0.334	0.044	8.38E-123

**cluster**	**order**	**gene**	**p_val**	**avg_logFC**	**pct.1**	**pct.2**	**p_val_adj**
**7**	1	*Car2*	0.00E+00	2.8052	0.975	0.148	0.00E+00
**7**	2	*Hmbs*	0.00E+00	2.6686	0.945	0.041	0.00E+00
**7**	3	*Hist1h1b*	0.00E+00	2.5751	0.549	0.04	0.00E+00
**7**	4	*Slc4a1*	0.00E+00	2.5201	0.897	0.081	0.00E+00
**7**	5	*Prdx2*	1.99E-278	2.469	0.972	0.388	6.19E-274

7	6	*Tmcc2*	0.00E+00	2.4167	0.589	0.021	0.00E+00
7	7	*Mki67*	0.00E+00	2.302	0.667	0.057	0.00E+00
7	8	*Gypa*	0.00E+00	2.1962	0.885	0.103	0.00E+00
7	9	*Blvrb*	0.00E+00	2.1916	0.927	0.236	0.00E+00
7	10	*Rhd*	0.00E+00	2.1702	0.772	0.008	0.00E+00
7	84	*Hmgb2*	1.19E-71	1.1337	0.694	0.346	3.69E-67
7	106	*Stmn1*	1.25E-102	0.9878	0.373	0.072	3.88E-98
7	117	*Epb41*	1.05E-83	0.9477	0.762	0.337	3.25E-79
7	119	*Isg20*	3.63E-115	0.947	0.807	0.315	1.13E-110
7	133	*H2afz*	8.58E-32	0.9064	0.571	0.349	2.66E-27
7	175	*Fam46c*	7.08E-51	0.7843	0.707	0.35	2.20E-46
7	181	*Fam213a*	1.58E-43	0.7616	0.424	0.161	4.90E-39
7	200	*Ube2o*	6.06E-48	0.7235	0.456	0.173	1.88E-43
7	248	*Snca*	1.30E-65	0.6561	0.842	0.395	4.03E-61
7	265	*Alas2*	9.37E-53	0.6263	0.865	0.463	2.91E-48
7	323	*Hbb-bt*	7.12E-42	0.5302	0.992	0.584	2.21E-37
7	332	*Ube2l6*	1.16E-33	0.5184	0.689	0.371	3.60E-29
7	392	*Mkrn1*	2.18E-25	0.4394	0.835	0.569	6.77E-21
7	475	*Isca1*	1.77E-23	0.3105	0.356	0.16	5.49E-19
7	509	*Fam220a*	9.39E-14	0.2733	0.584	0.369	2.92E-09

**cluster**	**order**	**gene**	**p_val**	**avg_logFC**	**pct.1**	**pct.2**	**p_val_adj**
**8**	1	*Ccl5*	0.00E+00	2.8793	0.308	0.004	0.00E+00
**8**	2	*Cd3g*	0.00E+00	2.7169	0.838	0.002	0.00E+00
**8**	3	*Cd3d*	0.00E+00	2.3614	0.826	0.009	0.00E+00
**8**	4	*Trbc2*	0.00E+00	2.2912	0.843	0.008	0.00E+00
**8**	5	*Lgals1*	1.10E-218	2.1323	0.624	0.101	3.41E-214
**8**	6	*AW112010*	0.00E+00	2.1146	0.738	0.041	0.00E+00
**8**	7	*Trdc*	0.00E+00	2.1102	0.259	0.001	0.00E+00
**8**	8	*S100a4*	2.35E-200	2.025	0.547	0.081	7.30E-196
**8**	9	*Ms4a4b*	0.00E+00	2.0039	0.536	0.003	0.00E+00
**8**	10	*H2-K1*	9.12E-297	1.8895	0.96	0.221	2.83E-292
**8**	139	*Ctla2a*	1.28E-166	1.0647	0.425	0.052	3.98E-162
**8**	564	*H2afz*	1.21E-48	0.5118	0.744	0.344	3.76E-44
**8**	725	*Fgl2*	5.70E-69	0.4347	0.316	0.065	1.77E-64

cluster	order	gene	p_val	avg_logFC	pct.1	pct.2	p_val_adj
**9**	1	*Fabp5*	0.00E+00	4.6917	0.901	0.124	0.00E+00
**9**	2	*Mt4*	0.00E+00	3.9902	0.492	0.018	0.00E+00
**9**	3	*Lgals7*	0.00E+00	3.828	0.924	0.029	0.00E+00
**9**	4	*Gsto1*	0.00E+00	2.9759	0.772	0.047	0.00E+00
**9**	5	*Krt14*	0.00E+00	2.8359	0.736	0.012	0.00E+00
**9**	6	*Krt6a*	0.00E+00	2.8126	0.673	0.01	0.00E+00
**9**	7	*Hspb1*	0.00E+00	2.7549	0.858	0.052	0.00E+00
**9**	8	*Fam162a*	0.00E+00	2.6598	0.809	0.07	0.00E+00
**9**	9	*Krt17*	0.00E+00	2.6484	0.571	0.009	0.00E+00
**9**	10	*Sfn*	0.00E+00	2.6339	0.779	0.017	0.00E+00
**9**	67	*Tpm2*	0.00E+00	1.6655	0.667	0.03	0.00E+00
**9**	147	*Ly6d*	1.49E-222	1.1676	0.393	0.029	4.61E-218
**9**	280	*Fxyd3*	5.30E-270	0.8281	0.383	0.021	1.64E-265
**9**	390	*Tpm1*	9.57E-102	0.6723	0.452	0.088	2.97E-97
**9**	466	*Stmn1*	1.60E-48	0.5994	0.317	0.077	4.97E-44

**cluster**	**order**	**gene**	**p_val**	**avg_logFC**	**pct.1**	**pct.2**	**p_val_adj**
**10**	1	*Ccl21a*	0.00E+00	4.2395	0.746	0.004	0.00E+00
**10**	2	*Mmrn1*	0.00E+00	3.6658	0.993	0.003	0.00E+00
**10**	3	*Lyve1*	0.00E+00	3.4541	0.804	0.005	0.00E+00
**10**	4	*Igfbp5*	0.00E+00	3.2626	0.851	0.044	0.00E+00
**10**	5	*Timp3*	0.00E+00	3.2036	0.975	0.044	0.00E+00
**10**	6	*Cldn5*	0.00E+00	3.1977	0.978	0.007	0.00E+00
**10**	7	*Fgl2*	0.00E+00	2.9448	0.931	0.05	0.00E+00
**10**	8	*Cavin2*	0.00E+00	2.5556	0.917	0.032	0.00E+00
**10**	9	*Gng11*	0.00E+00	2.4836	0.942	0.043	0.00E+00
**10**	10	*Sptbn1*	0.00E+00	2.4217	0.971	0.149	0.00E+00
**10**	19	*Ctla2a*	0.00E+00	2.1758	0.851	0.043	0.00E+00
**10**	24	*Tm4sf1*	0.00E+00	2.0438	0.678	0.035	0.00E+00
**10**	40	*Egfl7*	0.00E+00	1.8363	0.826	0.028	0.00E+00
**10**	55	*Pecam1*	0.00E+00	1.6244	0.859	0.034	0.00E+00
**10**	229	*Serping1*	0.00E+00	0.9738	0.612	0.045	0.00E+00
**10**	296	*H2-K1*	1.92E-79	0.8962	0.717	0.233	5.97E-75
**10**	519	*Ptprb*	3.72E-242	0.7103	0.366	0.02	1.15E-237
**10**	755	*Psap*	8.61E-89	0.5901	0.754	0.227	2.68E-84
**10**	1196	*Apoe*	2.15E-30	0.4367	0.297	0.091	6.69E-26

**cluster**	**order**	**gene**	**p_val**	**avg_logFC**	**pct.1**	**pct.2**	**p_val_adj**

**11**	1	*Igkc*	0.00E+00	5.496	0.802	0.03	0.00E+00
**11**	2	*Ighm*	0.00E+00	3.9795	0.885	0.043	0.00E+00
**11**	3	*Iglc1*	0.00E+00	3.6015	0.328	0	0.00E+00
**11**	4	*Cd79a*	0.00E+00	3.5534	0.927	0	0.00E+00
**11**	5	*Iglc2*	0.00E+00	3.2872	0.546	0.001	0.00E+00
**11**	6	*Ly6d*	0.00E+00	3.1772	0.752	0.021	0.00E+00
**11**	7	*Cd74*	0.00E+00	3.0573	0.71	0.035	0.00E+00
**11**	8	*Ebf1*	0.00E+00	3.0238	0.79	0.056	0.00E+00
**11**	9	*Vpreb3*	0.00E+00	2.9633	0.523	0.001	0.00E+00
**11**	10	*Cd79b*	0.00E+00	2.8537	0.748	0.004	0.00E+00
**11**	12	*H2-Aa*	0.00E+00	2.4219	0.557	0.026	0.00E+00
**11**	14	*H2-Ab1*	0.00E+00	2.2321	0.611	0.029	0.00E+00
**11**	16	*H2-Eb1*	0.00E+00	2.1231	0.55	0.024	0.00E+00
**11**	73	*H2-K1*	1.55E-67	1.4335	0.622	0.236	4.82E-63
**11**	290	*Ctss*	2.05E-38	0.6839	0.267	0.063	6.36E-34
**11**	327	*Plac8*	5.74E-08	0.5986	0.267	0.16	1.78E-03
**11**	342	*Hmgb2*	6.95E-01	0.5574	0.347	0.36	1.00E+00
**11**	347	*H2afz*	3.25E-02	0.5491	0.374	0.357	1.00E+00

**cluster**	**order**	**gene**	**p_val**	**avg_logFC**	**pct.1**	**pct.2**	**p_val_adj**
**12**	1	*Fabp4*	0.00E+00	3.2212	0.539	0.019	0.00E+00

**12**	2	*Mgp*	0.00E+00	2.936	0.65	0.024	0.00E+00
**12**	3	*Ctla2a*	0.00E+00	2.8107	0.78	0.046	0.00E+00
**12**	4	*Igfbp7*	1.94E-230	2.595	0.528	0.049	6.03E-226
**12**	5	*Tm4sf1*	0.00E+00	2.4388	0.791	0.034	0.00E+00
**12**	6	*Pecam1*	0.00E+00	2.4111	0.795	0.038	0.00E+00
**12**	7	*Vwf*	0.00E+00	2.4081	0.669	0.005	0.00E+00
**12**	8	*Sparc*	0.00E+00	2.3261	0.693	0.048	0.00E+00
**12**	9	*Egfl7*	0.00E+00	2.2837	0.772	0.031	0.00E+00
**12**	10	*Ptprb*	0.00E+00	2.2568	0.728	0.012	0.00E+00
**12**	18	*Gng11*	0.00E+00	1.9336	0.72	0.05	0.00E+00
**12**	22	*Sptbn1*	1.98E-209	1.86	0.827	0.154	6.15E-205
**12**	48	*Cavin2*	0.00E+00	1.5016	0.587	0.042	0.00E+00
**12**	62	*Hspb1*	1.01E-186	1.3927	0.555	0.064	3.12E-182
**12**	89	*Timp3*	5.22E-232	1.2266	0.587	0.056	1.62E-227
**12**	99	*Apoe*	2.21E-149	1.1712	0.567	0.085	6.86E-145
**12**	194	*Tpm1*	1.35E-109	0.9019	0.496	0.089	4.20E-105
**12**	231	*Plpp3*	1.14E-198	0.8541	0.539	0.055	3.53E-194
**12**	378	*H2-K1*	3.82E-50	0.7278	0.626	0.236	1.19E-45
**12**	575	*Igfbp4*	7.43E-87	0.5846	0.425	0.074	2.31E-82
**12**	984	*Ebf1*	4.02E-99	0.3779	0.433	0.066	1.25E-94
**12**	1138	*Psap*	4.46E-31	0.2845	0.559	0.233	1.39E-26

**cluster**	**order**	**gene**	**p_val**	**avg_logFC**	**pct.1**	**pct.2**	**p_val_adj**
**13**	1	*Camp*	2.72E-229	2.6261	1	0.216	8.43E-225
**13**	2	*Chil3*	3.24E-242	2.484	0.995	0.197	1.01E-237
**13**	3	*Ngp*	3.22E-221	2.2936	1	0.208	1.00E-216
**13**	4	*Hmgb2*	3.00E-176	2.2073	1	0.346	9.32E-172
**13**	5	*Hmgn2*	2.96E-271	2.1329	0.995	0.176	9.20E-267
**13**	6	*H2afz*	1.79E-172	2.0978	1	0.344	5.57E-168
**13**	7	*Serpinb1a*	2.44E-264	1.8599	0.972	0.149	7.58E-260
**13**	8	*Lcn2*	9.96E-170	1.8511	0.995	0.267	3.09E-165
**13**	9	*Ltf*	4.87E-247	1.7681	0.986	0.138	1.51E-242
**13**	10	*Stmn1*	0.00E+00	1.7229	0.944	0.066	0.00E+00
**13**	11	*Hist1h1b*	0.00E+00	1.7034	0.836	0.044	0.00E+00

**13**	14	*S100a9*	8.27E-103	1.5744	1	0.444	2.57E-98
**13**	18	*S100a8*	2.56E-100	1.5326	1	0.434	7.95E-96
**13**	22	*Mki67*	0.00E+00	1.424	0.935	0.062	0.00E+00
**13**	25	*Wfdc21*	4.30E-147	1.3742	0.991	0.222	1.33E-142
**13**	91	*Lyz2*	1.10E-109	0.9317	0.995	0.275	3.42E-105
**13**	163	*Ifitm6*	1.99E-117	0.7349	0.977	0.219	6.19E-113
**13**	363	*Plac8*	1.77E-96	0.507	0.729	0.151	5.48E-92

**cluster**	**order**	**gene**	**p_val**	**avg_logFC**	**pct.1**	**pct.2**	**p_val_adj**
**14**	1	*Lipf*	2.92E-63	6.8153	0.833	0.18	9.07E-59
**14**	2	*Sbpl*	4.10E-73	6.1474	0.972	0.264	1.27E-68
**14**	3	*Wfdc18*	3.52E-127	6.1158	0.986	0.132	1.09E-122
**14**	4	*2310057J18Rik*	3.25E-194	5.5834	0.972	0.076	1.01E-189
**14**	5	*Bpifb1*	2.78E-118	5.4949	0.944	0.128	8.63E-114
**14**	6	*Amy1*	1.14E-166	5.4836	0.972	0.09	3.54E-162
**14**	7	*Dcpp3*	8.86E-09	5.2994	0.361	0.152	2.75E-04
**14**	8	*Dcpp2*	5.43E-11	5.2414	0.375	0.14	1.69E-06
**14**	9	*Dcpp1*	6.22E-14	5.2379	0.347	0.101	1.93E-09
**14**	10	*Bpifa2*	1.34E-180	4.5083	0.792	0.05	4.15E-176
**14**	15	*Fxyd3*	7.77E-227	1.592	0.694	0.027	2.41E-222
**14**	21	*Wfdc2*	1.09E-95	1.1938	0.486	0.032	3.37E-91
**14**	29	*Nupr1*	6.30E-55	0.7189	0.708	0.116	1.96E-50

**14**	65	*H2-K1*	1.91E-14	0.4318	0.653	0.243	5.94E-10

**cluster**	**order**	**gene**	**p_val**	**avg_logFC**	**pct.1**	**pct.2**	**p_val_adj**
**15**	1	*Tff2*	3.23E-99	6.0946	1	0.177	1.00E-94
**15**	2	*Muc5b*	7.10E-76	5.4813	0.956	0.216	2.20E-71
**15**	3	*Bpifb2*	9.30E-254	5.0452	0.926	0.046	2.89E-249
**15**	4	*Nupr1*	6.91E-140	4.8635	1	0.114	2.15E-135
**15**	5	*Agr2*	1.52E-292	4.7664	0.971	0.044	4.71E-288
**15**	6	*Gp2*	0.00E+00	3.8439	0.838	0.011	0.00E+00
**15**	7	*Wfdc2*	0.00E+00	3.7074	0.853	0.03	0.00E+00
**15**	8	*Fxyd3*	0.00E+00	3.1705	0.897	0.026	0.00E+00
**15**	9	*Tesc*	8.05E-290	2.772	0.809	0.029	2.50E-285
**15**	10	*Smgc*	0.00E+00	2.6877	0.75	0.007	0.00E+00
**15**	22	*Gsto1*	1.71E-115	2.0984	0.735	0.064	5.29E-111
**15**	34	*AW112010*	3.77E-112	1.8788	0.721	0.06	1.17E-107
**15**	121	*Sbpl*	3.64E-07	1.3402	0.485	0.268	1.13E-02
**15**	347	*Isg20*	5.84E-08	0.9249	0.588	0.332	1.81E-03
**15**	475	*H2-K1*	2.88E-08	0.7785	0.5	0.244	8.95E-04

cluster	order	gene	p_val	avg_logFC	pct.1	pct.2	p_val_adj
**16**	1	*Acta1*	5.64E-179	5.6089	1	0.047	1.75E-174
**16**	2	*Tnnc2*	5.87E-294	5.4455	1	0.026	1.82E-289
**16**	3	*Mylpf*	6.09E-180	5.2576	1	0.046	1.89E-175
**16**	4	*Tnni2*	3.51E-242	4.9991	0.949	0.029	1.09E-237
**16**	5	*Tnnt3*	8.79E-278	4.9315	0.949	0.025	2.73E-273
**16**	6	*Ckm*	9.00E-272	4.8803	0.949	0.025	2.80E-267
**16**	7	*Myl1*	0.00E+00	4.7277	0.923	0.014	0.00E+00
**16**	8	*Tpm2*	3.74E-173	4.5059	0.974	0.046	1.16E-168
**16**	9	*Tpm1*	1.94E-76	4.457	0.897	0.096	6.04E-72
**16**	10	*Mb*	0.00E+00	4.4245	0.846	0.014	0.00E+00
**16**	219	*Lgals1*	2.96E-05	1.1398	0.333	0.118	9.18E-01
**16**	224	*Fam162a*	1.20E-04	1.1099	0.256	0.091	1.00E+00
**16**	252	*Sbpl*	2.59E-02	0.8756	0.103	0.27	1.00E+00
**16**	268	*Isca1*	7.80E-02	0.7727	0.256	0.167	1.00E+00
**16**	325	*Hspb1*	2.30E-05	0.4166	0.256	0.075	7.14E-01

Supplemental Table 6.

Top Five Genes in the Seventh and Ninth Cell Subtypes With Significant Differences Between the 29- and 16-Week Experimental Groups

Cluster7:29w treat vs.16w treat

	p_val	avg_logFC	pct.1	pct.2	p_val_adj
mt-Nd2	1.41E-09	2.847555	0.933	0.312	4.38E-05
Gm42418	2.76E-08	1.519975	0.94	0.875	0.000857
mt-Nd4l	5.49E-08	1.861324	0.817	0.062	0.001706
mt-Cytb	1.01E-07	1.875175	0.944	0.625	0.00313
mt-Nd3	2.03E-07	0.868717	0.908	0.188	0.006301

Cluster9: 29w treat vs. 16w treat

	p_val	avg_logFC	pct.1	pct.2	p_val_adj
Lnx1	6.06E-15	-0.4813	0	0.222	1.88E-10
Natd1	6.06E-15	-0.4813	0	0.222	1.88E-10
Gpr89	6.06E-15	-0.53658	0	0.222	1.88E-10
Krtap3-2	6.06E-15	-2.93493	0	0.222	1.88E-10
2300002M23Rik	9.23E-15	-3.09765	0.015	0.444	2.87E-10

Supplemental Table 7.

Genes in the Seventh Subtype Involved in the MYC_targets_v1 Pathway in the Experimental Group According to Gene Function Enrichment Analysis

PROBE	GENE SYMBOL	GENE_ TITLE	RANK IN GENE LIST	RANK METRIC SCORE	RUNNING ES	CORE ENRICHMENT
**RPS6**	null	null	7	1.921929	0.023955	Yes
**RPL14**	null	null	16	1.717817	0.043869	Yes
**RPS5**	null	null	18	1.688999	0.069343	Yes
**RPS2**	null	null	20	1.622016	0.093772	Yes
**RPS10**	null	null	26	1.541237	0.113509	Yes
**RPLP0**	null	null	30	1.471963	0.133882	Yes
**RPL18**	null	null	39	1.404468	0.148911	Yes
**PPIA**	null	null	65	1.167021	0.145647	Yes
**EEF1B2**	null	null	67	1.160469	0.16288	Yes
**RPL6**	null	null	68	1.142827	0.180697	Yes
**RPL34**	null	null	81	1.045431	0.186696	Yes
**NPM1**	null	null	82	1.043903	0.20297	Yes
**HSP90AB1**	null	null	87	1.017407	0.215399	Yes
**RACK1**	null	null	100	0.947806	0.219875	Yes
**RPL22**	null	null	107	0.917201	0.229024	Yes
**HNRNPD**	null	null	115	0.863213	0.236473	Yes
**NAP1L1**	null	null	116	0.857354	0.24984	Yes
**RPS3**	null	null	119	0.829343	0.261053	Yes
**PCNA**	null	null	123	0.804367	0.271018	Yes
**EIF4A1**	null	null	127	0.787659	0.280723	Yes
**SNRPD1**	null	null	129	0.781525	0.292048	Yes
**MCM7**	null	null	132	0.777428	0.302452	Yes
**DUT**	null	null	137	0.761807	0.310895	Yes
**HNRNPA3**	null	null	142	0.738997	0.318983	Yes
**NME1**	null	null	149	0.709877	0.3249	Yes
**CNBP**	null	null	152	0.705802	0.334187	Yes
**PSMA7**	null	null	165	0.665208	0.334258	Yes
**SET**	null	null	166	0.662229	0.344582	Yes
**NDUFAB1**	null	null	168	0.656574	0.35396	Yes
**RAN**	null	null	169	0.655036	0.364172	Yes
**U2AF1**	null	null	172	0.650413	0.372595	Yes
**RANBP1**	null	null	177	0.648398	0.379271	Yes
**EIF2S2**	null	null	180	0.647028	0.387641	Yes

**CCT7**	null	null	189	0.630371	0.390602	Yes
**GLO1**	null	null	191	0.622106	0.399443	Yes
**SNRPD2**	null	null	213	0.579745	0.390455	Yes
**PSMA2**	null	null	216	0.578158	0.397752	Yes
**HNRNPA1**	null	null	218	0.573356	0.405833	Yes
**CCT2**	null	null	219	0.573042	0.414767	Yes
**PCBP1**	null	null	223	0.565737	0.421011	Yes
**PSMD8**	null	null	236	0.539554	0.419123	Yes
**TCP1**	null	null	245	0.524732	0.420437	Yes
**HSPE1**	null	null	247	0.523499	0.42774	Yes
**SERBP1**	null	null	253	0.517677	0.431519	Yes
**CYC1**	null	null	257	0.513842	0.436955	Yes
**PSMD7**	null	null	258	0.512522	0.444945	Yes
**PRDX3**	null	null	262	0.502667	0.450207	Yes
**MCM2**	null	null	269	0.494272	0.452762	Yes
**SLC25A3**	null	null	275	0.488874	0.456092	Yes
**MCM6**	null	null	281	0.481197	0.459302	Yes
**EIF4H**	null	null	290	0.472741	0.459806	Yes
**MCM5**	null	null	304	0.463321	0.45587	Yes
**SRSF7**	null	null	305	0.46248	0.46308	Yes
**EIF3B**	null	null	323	0.446794	0.455454	Yes
**YWHAE**	null	null	324	0.446039	0.462408	Yes
**PABPC1**	null	null	330	0.443984	0.465038	Yes
**PHB2**	null	null	333	0.442072	0.470213	Yes
**RFC4**	null	null	337	0.435477	0.474427	Yes
**NHP2**	null	null	346	0.428529	0.474241	Yes
**SSB**	null	null	350	0.426064	0.478308	Yes
**PSMB2**	null	null	371	0.412382	0.46757	No
**HNRNPU**	null	null	380	0.40774	0.46706	No
**KPNB1**	null	null	401	0.394307	0.45604	No
**COX5A**	null	null	408	0.388835	0.456952	No
**YWHAQ**	null	null	429	0.378294	0.445682	No
**MCM4**	null	null	439	0.373637	0.443782	No
**CCT5**	null	null	443	0.371106	0.446993	No
**HNRNPC**	null	null	447	0.370191	0.450189	No
**CANX**	null	null	456	0.36708	0.449045	No
**EIF4G2**	null	null	460	0.366306	0.452181	No
**ETF1**	null	null	463	0.365634	0.456164	No
**USP1**	null	null	504	0.346987	0.427239	No
**PABPC4**	null	null	554	0.330141	0.390326	No
**C1QBP**	null	null	560	0.327777	0.391144	No
**HSPD1**	null	null	564	0.326982	0.393667	No

**EIF3D**	null	null	581	0.32289	0.384967	No
**ACP1**	null	null	582	0.322836	0.39	No
**EIF1AX**	null	null	584	0.322747	0.394174	No
**SNRPG**	null	null	589	0.320656	0.395739	No
**CCT3**	null	null	624	0.310227	0.371391	No
**NOP56**	null	null	630	0.307446	0.371892	No
**SNRPA1**	null	null	643	0.300314	0.366274	No
**UBA2**	null	null	653	0.295413	0.363154	No
**PRDX4**	null	null	658	0.292359	0.364279	No
**TARDBP**	null	null	665	0.290024	0.36365	No
**EIF2S1**	null	null	667	0.289879	0.367311	No
**SF3B3**	null	null	684	0.284587	0.358014	No
**LSM2**	null	null	688	0.284135	0.359869	No
**PSMC4**	null	null	712	0.277822	0.344458	No
**IMPDH2**	null	null	724	0.275129	0.339305	No
**CDK2**	null	null	762	0.267001	0.311708	No
**PTGES3**	null	null	763	0.266373	0.315861	No
**ERH**	null	null	774	0.263113	0.311379	No
**DEK**	null	null	844	-0.28217	0.256551	No
**PSMA4**	null	null	910	-0.41634	0.207248	No
**RRM1**	null	null	936	-0.49423	0.193494	No
**PSMD3**	null	null	990	-0.60764	0.157473	No
**TYMS**	null	null	1008	-0.65224	0.15305	No
**MAD2L1**	null	null	1091	-0.79078	0.094992	No
**HNRNPR**	null	null	1133	-0.84623	0.072992	No
**TRA2B**	null	null	1225	-1.24548	0.014298	No
**GNL3**	null	null	1233	-1.28523	0.028326	No

Supplemental Table 8.

Genes in the Ninth Subtype Involved in the MYC_targets_v1 Pathway in the Experimental Group According to Gene Function Enrichment Analysis

PROBE	GENE SYMBOL	GENE_ TITLE	RANK IN GENE LIST	RANK METRIC SCORE	RUNNING ES	CORE ENRICHMENT
**NPM1**	null	null	11	1.81545	0.017986	Yes
**SNRPD2**	null	null	26	1.381676	0.029435	Yes
**PPIA**	null	null	27	1.357527	0.046157	Yes
**SERBP1**	null	null	31	1.31939	0.061216	Yes
**NDUFAB1**	null	null	35	1.294732	0.075971	Yes
**RACK1**	null	null	38	1.265074	0.090759	Yes
**EIF4A1**	null	null	64	1.14563	0.094923	Yes
**PSMB2**	null	null	65	1.141965	0.10899	Yes
**PSMA7**	null	null	73	1.114053	0.119927	Yes
**HNRNPA3**	null	null	83	1.057133	0.129368	Yes
**CCT5**	null	null	91	1.035708	0.139341	Yes
**U2AF1**	null	null	117	0.969301	0.141333	Yes
**RPS2**	null	null	119	0.958663	0.152744	Yes
**SNRPD1**	null	null	121	0.954391	0.164102	Yes
**CCT2**	null	null	125	0.94974	0.174608	Yes
**EIF1AX**	null	null	148	0.908348	0.177042	Yes
**HNRNPA2B1**	null	null	167	0.877369	0.180687	Yes
**SRSF7**	null	null	175	0.866202	0.188572	Yes
**C1QBP**	null	null	183	0.854403	0.196311	Yes
**EIF4G2**	null	null	187	0.845553	0.205533	Yes
**HSPD1**	null	null	197	0.827542	0.212146	Yes
**LDHA**	null	null	198	0.8259	0.222319	Yes
**SNRPD3**	null	null	206	0.805131	0.229452	Yes
**ACP1**	null	null	212	0.796114	0.237269	Yes
**PSMA1**	null	null	226	0.781197	0.241719	Yes
**TCP1**	null	null	234	0.778243	0.24852	Yes
**HNRNPD**	null	null	237	0.773912	0.257257	Yes
**HSP90AB1**	null	null	242	0.76801	0.265126	Yes
**CCT4**	null	null	257	0.747694	0.268765	Yes
**RPS3**	null	null	267	0.739954	0.274299	Yes
**PSMA6**	null	null	273	0.733269	0.281342	Yes
**LSM2**	null	null	274	0.732965	0.290371	Yes

**MRPL23**	null	null	280	0.725712	0.297321	Yes
**RSL1D1**	null	null	296	0.706402	0.300053	Yes
**NME1**	null	null	298	0.704029	0.308328	Yes
**PSMD14**	null	null	312	0.696356	0.311733	Yes
**RANBP1**	null	null	313	0.695745	0.320303	Yes
**YWHAQ**	null	null	314	0.69489	0.328863	Yes
**PSMD7**	null	null	323	0.681654	0.334076	Yes
**RPLP0**	null	null	329	0.6783	0.340442	Yes
**EIF3B**	null	null	337	0.673092	0.345948	Yes
**PSMA4**	null	null	338	0.671935	0.354225	Yes
**RAD23B**	null	null	348	0.664518	0.358829	Yes
**RPS6**	null	null	360	0.654325	0.362512	Yes
**UBA2**	null	null	373	0.645528	0.365689	Yes
**GSPT1**	null	null	377	0.642436	0.372409	Yes
**FBL**	null	null	388	0.633723	0.376236	Yes
**SSBP1**	null	null	393	0.629844	0.382403	Yes
**SYNCRIP**	null	null	427	0.603662	0.376707	Yes
**PSMD1**	null	null	434	0.596355	0.381665	Yes
**RPS10**	null	null	453	0.584197	0.381699	Yes
**EIF2S1**	null	null	455	0.582564	0.388477	Yes
**DEK**	null	null	456	0.58122	0.395637	Yes
**CYC1**	null	null	475	0.56754	0.395465	Yes
**PSMC4**	null	null	477	0.567122	0.402053	Yes
**ABCE1**	null	null	499	0.55219	0.400499	Yes
**GNL3**	null	null	505	0.549246	0.405275	Yes
**COX5A**	null	null	515	0.544633	0.408402	Yes
**CBX3**	null	null	520	0.53989	0.413461	Yes
**CDK4**	null	null	567	0.518266	0.401541	Yes
**VDAC1**	null	null	569	0.517363	0.407516	Yes
**YWHAE**	null	null	574	0.516021	0.41228	Yes
**UBE2L3**	null	null	601	0.498216	0.408071	Yes
**PHB2**	null	null	603	0.495808	0.413781	Yes
**RNPS1**	null	null	613	0.492135	0.416262	Yes
**HNRNPU**	null	null	642	0.477076	0.410996	No
**NCBP2**	null	null	645	0.475643	0.41606	No
**HDAC2**	null	null	681	0.459876	0.407797	No
**PA2G4**	null	null	689	0.455037	0.410617	No
**RPL14**	null	null	696	0.452547	0.413804	No
**TRA2B**	null	null	709	0.446332	0.414527	No
**NAP1L1**	null	null	791	0.418849	0.387454	No
**TXNL4A**	null	null	797	0.41702	0.390601	No
**SRM**	null	null	821	0.409868	0.386498	No

**VBP1**	null	null	823	0.409172	0.39114	No
**ODC1**	null	null	831	0.406248	0.393359	No
**PSMA2**	null	null	871	0.394101	0.382694	No
**ERH**	null	null	943	0.371203	0.359013	No
**SNRPA1**	null	null	970	0.36377	0.353148	No
**SRPK1**	null	null	979	0.361058	0.354412	No
**CCT7**	null	null	1004	0.352656	0.349206	No
**RPL18**	null	null	1017	0.349581	0.348737	No
**CDC20**	null	null	1032	0.345605	0.347424	No
**KPNB1**	null	null	1050	0.340422	0.344852	No
**RPL34**	null	null	1051	0.34029	0.349044	No
**SNRPG**	null	null	1065	0.337591	0.348029	No
**UBE2E1**	null	null	1085	0.33174	0.344555	No
**HDGF**	null	null	1093	0.329806	0.345832	No
**BUB3**	null	null	1129	0.322303	0.335875	No
**RFC4**	null	null	1144	0.318195	0.334223	No
**XPOT**	null	null	1147	0.31756	0.337339	No
**IMPDH2**	null	null	1212	0.305263	0.315632	No
**HDDC2**	null	null	1235	0.301811	0.310595	No
**G3BP1**	null	null	1251	0.299018	0.30831	No
**APEX1**	null	null	1260	0.298259	0.3088	No
**GLO1**	null	null	1276	0.294869	0.306464	No
**COPS5**	null	null	1282	0.293796	0.308093	No
**MRPL9**	null	null	1285	0.292887	0.310905	No
**EIF3D**	null	null	1358	0.280467	0.285709	No
**FAM120A**	null	null	1368	0.279407	0.285569	No
**ILF2**	null	null	1375	0.277851	0.286605	No
**SMARCC1**	null	null	1379	0.276642	0.288818	No
**HSPE1**	null	null	1385	0.275385	0.290221	No
**TRIM28**	null	null	1405	0.272224	0.286014	No
**PPM1G**	null	null	1417	0.271045	0.284975	No
**SNRPB2**	null	null	1425	0.269277	0.285507	No
**DUT**	null	null	1433	0.26845	0.286028	No
**PABPC4**	null	null	1464	0.263897	0.277341	No
**AIMP2**	null	null	1513	0.255493	0.261388	No
**PSMD8**	null	null	1551	0.2511	0.249757	No
**SRSF3**	null	null	1672	-0.27793	0.205429	No
**SLC25A3**	null	null	1701	-0.28547	0.197804	No
**PSMB3**	null	null	1873	-0.35534	0.134135	No
**CANX**	null	null	1940	-0.39935	0.11279	No
**EIF2S2**	null	null	2032	-0.47492	0.082429	No
**SET**	null	null	2106	-0.54507	0.060094	No

**PABPC1**	null	null	2133	-0.59894	0.057126	No
**SSB**	null	null	2135	-0.60063	0.064127	No
**PCBP1**	null	null	2161	-0.6407	0.062071	No
**NHP2**	null	null	2190	-0.70586	0.059624	No
**PGK1**	null	null	2194	-0.7103	0.06718	No
**HNRNPA1**	null	null	2196	-0.71806	0.075627	No
**CNBP**	null	null	2230	-0.8301	0.072721	No
**PTGES3**	null	null	2257	-0.89594	0.073411	No
**CCT3**	null	null	2292	-0.99607	0.072151	No
**IFRD1**	null	null	2310	-1.02258	0.077983	No
**TFDP1**	null	null	2324	-1.06258	0.085899	No
**EIF4H**	null	null	2459	-1.3787	0.049559	No
**POLD2**	null	null	2574	-1.82379	0.026661	No
